# Critical role of keratinocytes and protease‐activated receptor 2 in secondary lymphedema development

**DOI:** 10.1002/ctm2.70682

**Published:** 2026-06-01

**Authors:** Hyeung Ju Park, Sarit Pal, Xizhao Chen, Jinyeon Shin, Gabriela D. García Nores, Jung Eun Baik, Annica Stull‐Lane, Abraham J. Book, Cristina C. Clement, Elizabeth M. Encarnacion, Mark G. Klang, Elyn Riedel, Tafadzwa L. Chaunzwa, Geoffrey E. Hespe, Laura Santambrogio, Michelle Coriddi, Joseph H. Dayan, Babak J. Mehrara, Raghu P. Kataru

**Affiliations:** ^1^ Plastic and Reconstructive Surgery Service Department of Surgery Memorial Sloan Kettering Cancer Center New York New York USA; ^2^ Advanced Computing and Oncology Laboratory Department of Radiation Oncology Memorial Sloan Kettering Cancer Center New York New York USA; ^3^ Department of Radiation Oncology Weill Cornell Medicine New York New York USA; ^4^ Research Pharmacy Core Memorial Sloan Kettering Cancer Center New York New York USA; ^5^ Biostatistics Service Department of Epidemiology and Biostatistics Memorial Sloan Kettering Cancer Center New York New York USA

**Keywords:** hyperkeratosis, keratinocyte, PAR2, secondary lymphedema, teriflunomide, Th2‐inducing cytokines, TSLP

## Abstract

**Background:**

Secondary lymphedema is a common complication of cancer treatment and epidermal changes are recognised as histological hallmarks of secondary lymphedema; however, the role of keratinocytes in the pathophysiology of this disease remains unclear.

**Methods:**

Hyperkeratosis, up‐regulation of protease‐activated receptor 2 (PAR2) and Th2‐inducing cytokines were assessed in biopsy specimens from patients with unilateral breast cancer‐related lymphedema (BCRL) and in a mouse model of lymphedema. PAR2 inhibition using global PAR2 knockout, keratinocyte‐specific PAR2 KO and bone marrow chimera models, or keratinocyte proliferation inhibition using a topical formulation of Teriflunomide (TF), was analysed in mouse models of lymphedema. We also assessed the direct effects of patient‐derived lymphedema lymph fluid (LF) on keratinocyte activation in vitro.

**Results:**

Hyperkeratosis, expression of Th2‐inducing cytokines and PAR2 were significantly increased in BCRL patient biopsies and mouse models. Keratinocytes play a primary role in the lymphedema development by producing T helper 2 (Th2)‐inducing cytokines. Specifically, keratinocyte proliferation and PAR2 expression are early responses following lymphatic injury and regulate the expression of Th2‐inducing cytokines, the migration of Langerhans cells and the infiltration of Th2‐differentiated T cells into the skin. Deficiency of PAR2 or topical inhibition of thymic stromal lymphopoietin rescues secondary lymphedema by reducing Th2 inflammation. Inhibition of PAR2 activation with a small‐molecule inhibitor, or the proliferation of the inhibitor TF, prevents activation of keratinocytes stimulated with lymphedema fluid. Finally, topical TF is highly effective in reducing swelling, fibrosis and inflammation and the overall pathology of lymphedema.

**Conclusions:**

Our findings suggest that lymphedema is a chronic inflammatory skin disease, and topically targeting keratinocyte inhibition may be a clinically effective therapy for this condition.

**Key points:**

- Activated keratinocytes play a key role in the pathophysiology of secondary lymphedema through PAR2 by producing Th2‐inducing cytokines that modulate skin inflammatory responses.

## Graphical abstract

Activated keratinocytes play an important role in the early stage of the pathophysiology of secondary lymphedema through PAR2 signaling. Lymphatic injury‐induced protease activation stimulates keratinocyte PAR2 signaling, driving hyperkeratosis and Th2 inflammation, which can be therapeutically attenuated by teriflunomide.

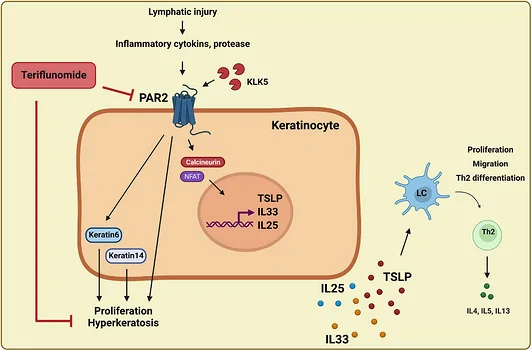

## 1 INTRODUCTION

Lymphedema is a chronic condition caused by inadequate lymphatic function, resulting in hyperkeratosis and fibroadipose deposition.^1^ In developed countries, the most common cause of lymphedema is lymph node excision during cancer surgery. It is estimated that 25–40% of patients who undergo surgical treatment for solid tumours develop lymphedema.^2^ Current treatments for secondary lymphedema – decongestive therapy or compression garments – are costly and palliative.^3^, ^4^ Likewise, although surgical treatments aimed at improving the development of collateral lymphatics are helpful in some patients, they are not effective for patients with advanced disease and can cause additional morbidity.^5^

Several lines of evidence suggest that the pathophysiology of lymphedema is related to chronic cutaneous T helper cell inflammatory responses.^6^, ^7^, ^8^, ^9^, ^10^, ^11^, ^12^, ^13^, ^14^ CD4^+^ T cell abundance is increased in clinical biopsy specimens, and this inflammatory response positively correlates with severity of disease.^15^ Depletion of CD4^+^ T cells (but not CD8^+^ cells, natural killer cells, macrophages or B cells) in mouse models prevents the development of lymphedema and effectively treats established disease.^6^, ^7^, ^8^, ^16^, ^17^ Topical delivery of tacrolimus, a drug that inhibits T cell proliferation, is highly effective for treating lymphedema in mouse models.^8^

Recent studies have shown that T helper 2 (Th2) inflammatory responses and arachidonic acid metabolites play an important role in the pathophysiology of lymphedema by promoting fibrosis and lymphatic leakiness and impairing the pumping function of collecting lymphatics.^3^, ^8^, ^13^, ^15^, ^16^, ^18^, ^19^ Th2 differentiation of naïve CD4^+^ cells is necessary for lymphedema development, as inhibition of this response with neutralising antibodies targeting IL‐4 or IL‐13 or in genetic models deficient in Th2 differentiation is effective for treating the disease.^15^, ^18^ In patients with breast cancer‐related lymphedema (BCRL), we found that once‐monthly infusions of IL‐4/IL‐13‐neutralising antibodies significantly improved histologic skin abnormalities and decreased the symptoms of the disease.^20^ These findings are supported by clinical trials and mouse studies demonstrating that doxycycline is effective for treating filariasis‐induced secondary lymphedema by decreasing Th2 immune responses.^21^, ^22^ Although Th2 inflammatory responses are necessary and sufficient for lymphedema development, how these responses are activated remains unclear, hindering the development of new therapies for this disease.

Epidermal changes are a prominent finding in lymphedema and include hyperkeratosis, acanthosis, spongiosis and parakeratosis with elongated rete edges.^23^, ^24^ These skin changes are similar to the epidermal changes in atopic dermatitis (AD).^25^ As with lymphedema, the pathology of AD is regulated by Th2 inflammatory responses. Importantly, epidermal changes in AD are a primary event and precede infiltration of Th2 cells in the skin. Keratinocytes regulate Th2 inflammatory responses in AD by producing Th2‐inducing cytokines, such as protease‐activated receptor 2 (PAR2), thymic stromal lymphopoietin (TSLP), IL‐33 and IL‐25.^26^, ^27^, ^28^, ^29^, ^30^ These Th2‐inducing cytokines act on naïve CD4^+^ cells through activation of dendritic cells (DCs), type 2 innate lymphoid cells (ILC2), to prime Th2 differentiation, regulate cytokine and migratory responses of antigen‐presenting cells (APCs) and stimulate proliferation of granulocytes like basophils, mast cells that release Th2 cytokines.^31^, ^32^, ^33^ The importance of a Th2 response in AD is highlighted by the efficacy of dupilumab, a monoclonal antibody that prevents IL‐4/IL‐13 signalling.^34^ Thus, the parallels between AD and lymphedema suggest that keratinocytes may play an important role in the pathophysiology of secondary lymphedema.

In this study, we tested this hypothesis using clinical lymphedema biopsy specimens and mouse models. We show that lymphatic injury results in increased expression of PAR2 and Th2‐inducing cytokines by keratinocytes and that this response is regulated by exposure to lymphatic fluid (LF). Inhibition of PAR2 activation or of keratinocyte proliferation attenuates the expression of Th2‐inducing cytokines and is effective in preventing lymphedema development in mouse models.

## 2 RESULTS

### 2.1 Lymphedema results in hyperkeratosis, de‐differentiation of epidermal cells and increased expression of Th2‐inducing cytokines, and state‐dependent expression of PAR2 in keratinocytes

**FIGURE 1:**
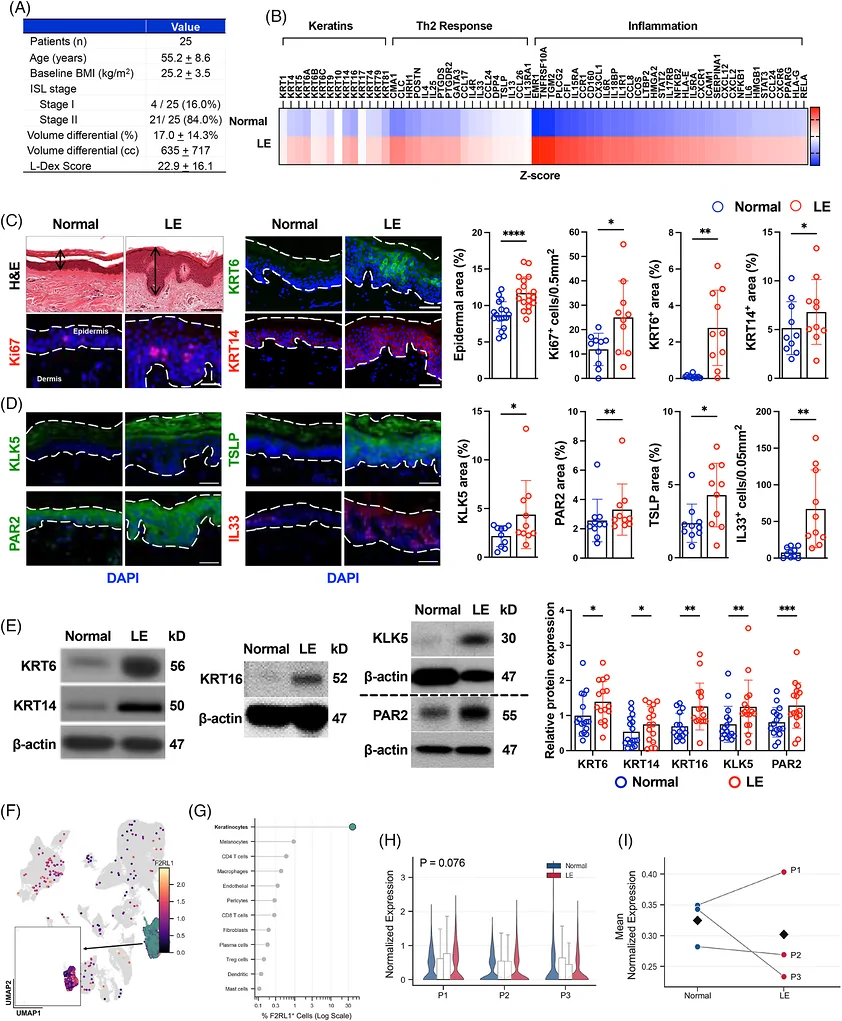
Lymphedema results in hyperkeratosis and expression of Th2‐inducing cytokines. (A) Demographic characteristics of patients with unilateral BCRL who provided samples for our study. Data are mean ± standard deviation unless noted. (B) Expression of keratins, Th2 response and inflammatory genes by RNAseq in normal and lymphedematous (LE) skin biopsies from patients with unilateral BCRL (*n* = 4). Each box represents the average mRNA expression of four patients. (C) Representative images (left) and quantification (right) of H&E and immunofluorescent staining of Ki67, KRT6 and KRT14 in normal and LE skin. Arrow indicates epidermis, and dashed lines indicate the thickness of epidermis. Scale bars: H&E, 100 µm; immunofluorescent staining, 50 µm. (Right) Each circle represents the average quantification of three high‐power field (HPF) views for each patient (*n *= 10). *p < .05, **p < .01, ****p < .0001. *p* Values were calculated by paired Student's *t*‐test. (D) Representative immunofluorescent images (left) and quantification (right) of KLK5, PAR2, TSLP and IL‐33 staining in normal and LE skin. Dashed lines indicate the thickness of epidermis. Scale bar: 50 µm. (Right) Each circle represents the average quantification of three HPF views for each patient (*n *= 10). *p < .05, **p < .01. *p* Values were calculated by Student's *t*‐test. (E) Representative western blots (left) and quantification (right; relative to β‐actin) of KRT6, KRT14, KRT16, KLK5 and PAR2 in normal and LE skin. (Right) Each circle represents one patient (*n *= 16). *p < .05, **p < .01, ***p < .001. *p* Values were calculated by paired Student's *t*‐test. (F) UMAP plot of single cells integrated from normal and LE skin. (G) Cross‐cell‐type specificity of percent F2RL1‐positive cells. (H) Normalised F2RL1/PAR2 expression in keratinocytes stratified by paired donor and condition. (I) Donor‐paired mean normalised F2RL1/PAR2 expression across all keratinocytes. *p* = .6782. *p* Values were calculated by paired Student's *t*‐test.

To analyse epidermal changes resulting from lymphedema, we analysed skin biopsy samples from the normal and lymphedematous arms of 25 patients with unilateral stage I–II upper extremity BCRL (Figure 1A). Bulk RNA sequencing (RNAseq) analysis showed evidence of a Th2 inflammatory response and increased expression of keratin genes in lymphedematous samples (KRT; Figure 1B). Notably, we found increased expression of KRT6, a keratin that is indicative of highly proliferative and activated keratinocytes commonly observed in pathological skin conditions,^35^ and KRT14, a keratin that is expressed by mitotically active, less differentiated keratinocytes typically found in the basal layer of the skin.^35^, ^36^ Using quantitative PCR (qPCR), we confirmed that KRT6, KRT14 and KRT16 expression was significantly increased in the lymphedematous arm compared with the normal arm, although there was some inter‐patient variability (Figure S1A). Histological analysis of skin biopsy samples confirmed that lymphedema was associated with hyperkeratosis, an increased epidermal area, an increased number of proliferating Ki67^+^ keratinocytes and increased expression of KRT6 and KRT14 (Figure 1C). KRT6‐expressing keratinocytes were enlarged and abnormal in appearance, and KRT14 expression was noted in all layers of the epidermis of lymphedematous skin, indicating decreased keratinocyte differentiation (Figure 1C).

PAR2 is a regulator of Th2‐inducing cytokines, such as TSLP and IL‐33 in AD. It is activated by serine proteases, such as kallikrein 5 (KLK5), which cleave N‐terminal of the PAR2 molecule to expose the tethered ligand.^37^ We found that the expression of PAR2, KLK5 and Th2‐inducing cytokines TSLP and IL‐33 was markedly increased in lymphedematous skin compared with normal skin (Figure 1D). KLK5 staining was localised to the cornified layer of the skin, whereas PAR2, TSLP and IL‐33 staining were present and increased in the entire epidermis (Figure 1D). We used negative (no primary antibody) controls to confirm the specificity of our findings (Figure S1B). Western blot analysis also confirmed the increased KRT6, KRT14, KRT16, KLK5 and PAR2 expression in lymphedematous skin (Figure 1E). Interestingly, bulk RNA‐seq analysis revealed variable expression of F2RL1/PAR2 and KLK5 at the individual level (Figure S2A).

PAR2 activates the expression of Th2‐inducing cytokines by NFATc1 activation.^38^ Consistent with this, we found that NFATc1 expression is significantly higher in lymphedematous skin than in normal skin. In addition, these keratinocyte changes correlated with higher expression of keratinocyte growth factors (epithelial growth factor [EGF], EGF receptor, IL‐1α) in lymphedematous skin (Figure S1C).

Because PAR2 is expressed by keratinocytes, immune cells and endothelial cells,^39^ we analysed single‐cell RNAseq data from the normal and lymphedematous skin of three lymphedema patients. Consistent with our histological findings, we observed that keratinocytes from lymphedematous samples showed enriched expression of F2RL1/PAR2, KRT6 and KRT14 compared with normal skin (Figure 1F,G). However, at the population level, the mean percentage of F2RL1‐positive keratinocytes showed a trend towards reduction in lymphedematous skin (41.6% normal vs. 36.4% LE, paired *t*‐test, *p* = .076) (Figure 1H,I). State‐level analysis revealed that this heterogeneity was driven by both shifts in keratinocyte state composition and state‐specific regulation of F2RL1 expression (Figure S2B,C). LE skin demonstrated a marked expansion of differentiating keratinocytes (K3) and a reduction in basal keratinocytes (K0) (Figure S2D). F2RL1 expression was not uniformly altered across all keratinocyte states. Proliferative/cycling keratinocytes (K6) and stress‐response keratinocytes (K8) showed marked reductions in F2RL1‐positive cell fractions in lymphedematous samples, while differentiated keratinocytes (K3) showed minimal to moderate change (Figure S2E). The overall reduction in F2RL1‐positive keratinocytes was driven primarily by within‐state expression changes (−4.62 percentage points) rather than compositional shifts (−.62 percentage points).

### 2.2 Th2‐inducing cytokine expression by keratinocytes increases rapidly after lymphatic injury and precedes T cell inflammatory responses

**FIGURE 2:**
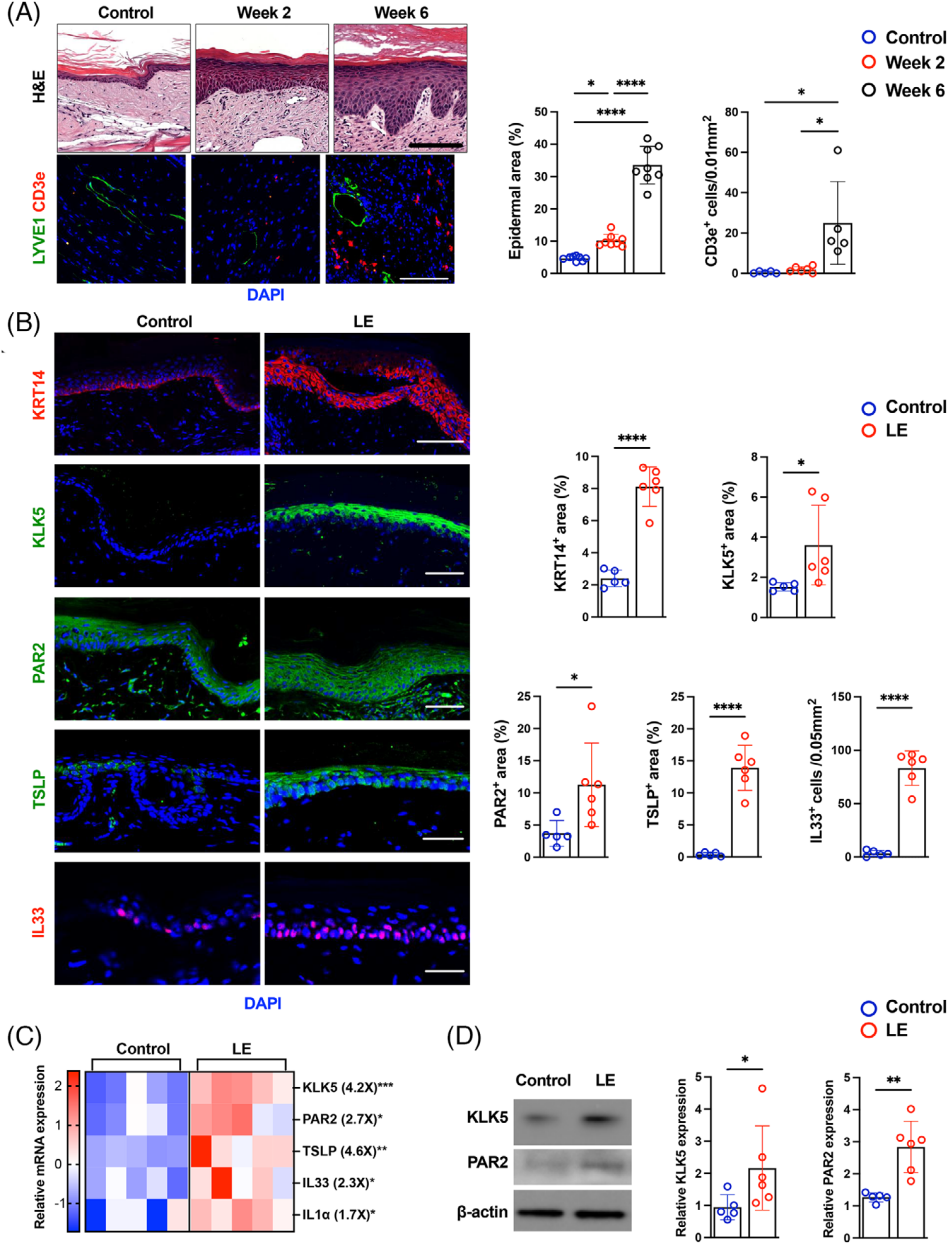
Keratinocyte expression of Th2‐inducing cytokines occurs rapidly after lymphatic injury. (A) Representative H&E (top) and immunofluorescent staining for LYVE1 and CD3e (bottom), and quantification (right) of control (skin incision) tail skin and tail skin harvested 2 or 6 week after skin and lymphatic excision. Scale bar: 100 µm. Each circle represents the average of three HPF views for each mouse (*n* = 5–8). *p < .05, ****p < .0001. *p* Values were calculated by one‐way ANOVA. (B) Representative immunofluorescent images (left) and quantification (right) of KRT14, KLK5, PAR2, TSLP, IL‐33, Ki67 and IL1α staining in tail skin harvested 2 week after surgery from control and lymphedema (LE) mice. Scale bar: 100 µm. Each circle represents the average of three HPF views for each mouse (*n *= 5–6). *p < .05, ****p < .0001. *p* Values were calculated by unpaired Student's *t*‐test. (C) Relative mRNA expression by qPCR tail skin harvested 2 week after surgery from control and LE mice (*n *= 5). mRNA expression was normalised to β‐actin expression. Each box represents one mouse. *p < .05, **p < .01, ***p < .001. *p* Values were calculated by Mann–Whitney test. Fold changes from control are shown in parentheses. (D) Representative western blot (left) and quantification (right; relative to β‐actin) of KLK5 and PAR2 in tail skin harvested 2 week after surgery from control and LE mice (left). Each circle represents each mouse (*n *= 5–6). *p < .05, **p < .01. *p* Values were calculated by Mann–Whitney test. (E) TSLP and IL‐33 ELISA from protein lysates of control and LE tail skin (*n *= 5–7). Each circle represents one mouse. *p* Values were calculated by Mann–Whitney test.

We next used a mouse tail model of lymphedema to understand the temporal changes in the epidermis relative to the timing of lymphatic injury and the development of lymphedema. In this model, histological signs of lymphedema, such as Th2 cell infiltration and fibroadipose deposition, develop 4–6 week after skin and lymphatic excision.^16^, ^40^ We therefore harvested tail skin specimens 2 and 6 week after surgery to analyse epidermal changes before and after the onset of lymphedema.^40^ We found that hyperkeratosis occurred rapidly after lymphatic injury and was evident even at the 2‐week time point in skin sections harvested 2–3 cm from the excision site (Figure 2A,B). Hyperkeratosis increased significantly by 6 week after surgery, suggesting that epidermal changes are progressive in nature. These epidermal changes preceded dermal infiltration of CD3^+^ T cells (Figure 2A).

At the 2‐week time point even before the onset of lymphedema, we also found that the expression of KRT14, KLK5, PAR2, TSLP, IL‐33, Ki67 and IL‐1α (Figure 2B) were increased in lymphedematous skin samples compared with control skin. We confirmed our histological findings with qPCR and found that the expression of KLK5 (4.2‐fold), PAR2 (2.7‐fold), TSLP (4.6‐fold), IL‐33 (2.3‐fold) and IL‐1α (1.7‐fold) were significantly increased in lymphedematous skin specimens (Figure 2C). Western blotting for KLK5 and PAR2 (Figure 2D) and ELISA for TSLP and IL‐33 (Figure 2E) showed that these mRNA changes translated to increased protein expression in lymphedematous skin.

### 2.3 PAR2 deficiency reduces Th2 inflammation and lymphedema

**FIGURE 3:**
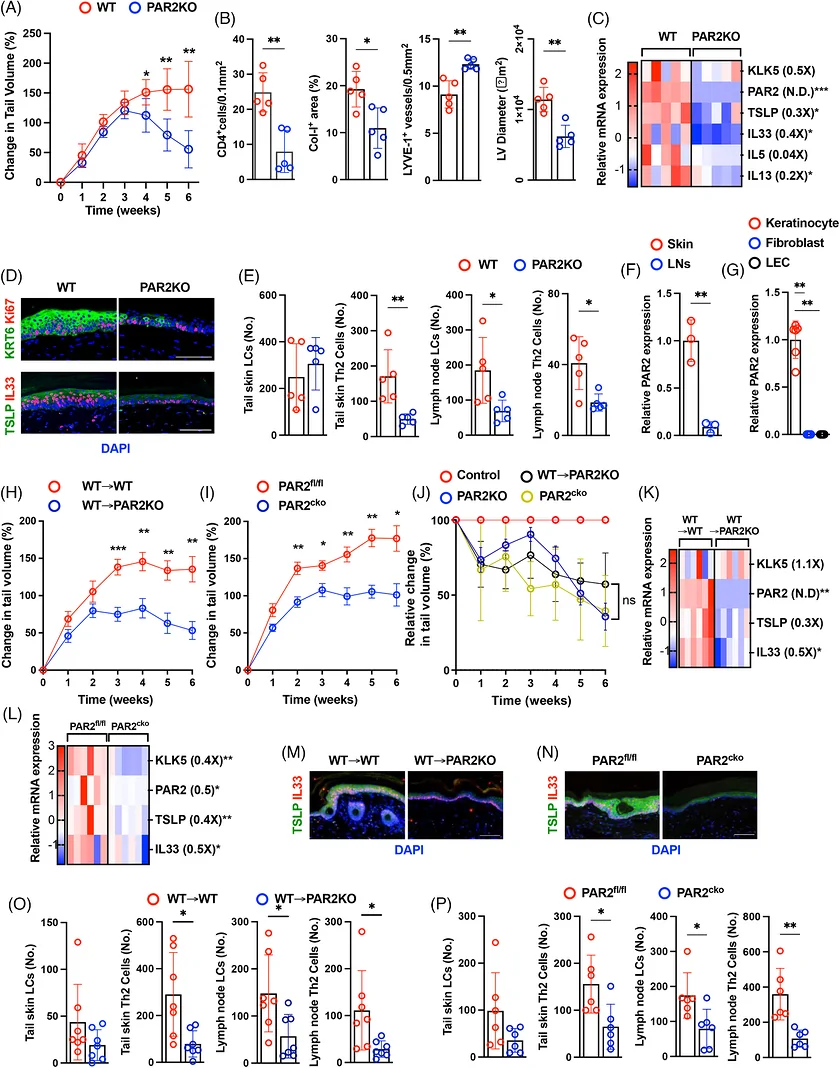
PAR2 deficiency of keratinocytes reduces lymphedema. (A) Changes in tail volume over time in WT and PAR2KO mice. Each circle represents the average measurement from each mouse (*n *= 5). *p < .05, **p < .01. *p* Values were calculated by two‐way ANOVA. (B) Quantification of CD4^+^ cells, collagen I positivity, LYVE1^+^ vessels and lymphatic vessel diameter in the tail skin of WT and PAR2KO mice. Each circle represents the average quantification of three HPF views for each mouse (*n *= 5). *p < .05, **p < .01. *p* Values were calculated by Mann–Whitney test. (C) Relative mRNA expression by qPCR in the tail skin of WT and PAR2KO mice (*n *= 5). mRNA expression was normalised by β‐actin expression. Each box represents one mouse. *p < .05, ***p < .001. *p* Values were calculated by Mann–Whitney test. Fold change from control is shown in parentheses. (D) Representative immunofluorescent images of KRT6, Ki67, TSLP and IL‐33 staining in the tail skin of WT and PAR2KO mice. Scale bar: 100 µm. (E) Quantification of the number of LCs and Th2 cells from the tail skin and draining lymph nodes of WT and PAR2KO mice. Each circle represents each mouse (*n *= 5). *p < .05, **p < .01. *p* Values were calculated by Mann–Whitney test. (F) Relative expression of PAR2 in the skin and lymph nodes (LNs). Each circle represents each mouse (*n *= 3). **p < .01. *p* Values were calculated by Mann–Whitney test. (G) Relative expression of PAR2 in keratinocytes, fibroblasts and lymphatic endothelial cells (LEC). Each circle represents each replicate (*n *= 6). ****p < .0001. *p* Values were calculated by two‐way ANOVA. (H and I) Changes in tail volume from WT→WT and WT→PAR2KO mice (H) or PAR2^fl/fl^ and PAR2^cko^ mice (I) 6 week after tail skin and lymphatic excision. Tail skin was harvested 6 week after surgery. Each circle represents the average measurement for each mouse (*n *= 6–9). *p < .05, **p < .01, ***p < .010. *p* Values were calculated by two‐way ANOVA. (J) Relative changes in tail volume over time in PAR2KO, WT→PAR2KO and PAR2^cko^ mice. Relative change in tail volume was calculated based on respective control of each group at each time point (PAR2KO/WT, WT→PAR2KO/ WT→WT and PAR2^cko^/ PAR2^fl/fl^). Each circle represents the average value from each mouse (*n *= 5). (K and L) Relative mRNA expression by qPCR in the tail skin of WT→WT and WT→PAR2KO mice (K) or PAR2^fl/fl^ and PAR2^cko^ mice (L) harvested 6 week after tail skin and lymphatic excision (*n *= 6). mRNA expression was normalised by β‐actin expression. Each box represents one mouse. *p < .05, **p < .01. *p* Values were calculated by Mann–Whitney test. Fold change from control is shown in parentheses. (M and N) Representative immunofluorescent images of TSLP and IL‐33 staining in the tail skin of WT→WT and WT→PAR2KO mice (M) or PAR2^fl/fl^ and PAR2^cko^ mice (N). Scale bar: 100 µm. (O and P) Quantification of the number of LCs and Th2 cells from the tail skin and draining lymph nodes of WT→WT and WT→PAR2KO mice (O) or PAR2^fl/fl^ and PAR2^cko^ mice (P). Each circle represents each mouse (*n *= 5). *p < .05, **p < .01. *p* Values were calculated by Mann–Whitney test.

PAR2 regulates the expression of Th2‐inducing cytokines and Th2 inflammatory responses in skin disorders. Inhibition of PAR2 decreases the severity of skin diseases such as AD and Netherton syndrome.^41^, ^42^ To investigate the role of PAR2 in lymphedema development, we compared swelling and secondary changes of lymphedema (inflammation, fibrosis and lymphatic dilatation) in WT and PAR2 knockout (PAR2KO) mice 6 week after tail skin and lymphatic excision. We noted an increase in tail volume in both WT and PAR2KO mice early after surgery (1–3 week; Figure 3A). However, compared with WT mice, tail swelling in PAR2KO mice decreased significantly thereafter; 6 week after surgery, PAR2KO mice had a twofold decrease in tail swelling (Figures 3A and S3A). Deletion of PAR2 resulted in significant tail oedema swelling and less dilated dermal LYVE‐1^+^ lymphatic vessels, suggesting better lymphatic drainage (Figure S3B). Furthermore, loss of PAR2 significantly decreased type I collagen deposition and CD4^+^ cell infiltration 6 week after surgery (Figures 3B and S3C). Consistent with a decreased lymphedema phenotype, PAR2KO mice also had an increased number of lymphatic vessels and a decreased lymphatic vessel diameter compared with WT controls (Figures 3C and S3C).

Gene expression analysis showed decreased expression of PAR2, TSLP, IL‐33, IL‐5 and IL‐13 in PAR2KO mice tail skin compared with WT (Figure 3C). Protein and histological analysis confirmed that PAR2 deletion resulted in decreased NFATc1 expression, whereas KLK5, upstream of PAR2, was not affected (Figure S3D,E). Loss of PAR2 also decreased hyperkeratosis and expression of KRT6, Ki67, TSLP and IL‐33 relative to controls (Figures 3D and S3F–H). Considering the importance of APCs in Th2 immune responses in lymphedema, we also examined the number of Langerhans cells (LCs) and Th2 cells in the skin and draining lymph nodes 6 week after tail skin and lymphatic excision. We found that loss of PAR2 did not significantly alter the number of activated LCs in the skin but decreased the number of LCs in the draining lymph nodes. PAR2KO mice also had significantly decreased numbers of Th2 cells infiltrating the skin and draining lymph nodes (Figures 3E and S3I).

Although we found that PAR2 expression is significantly higher in keratinocytes, several studies suggest that other cell types express PAR2.^43^, ^44^ We compared the expression of PAR2 in WT mice among tissues and noted that skin showed a higher expression compared with lymph nodes (Figure 3F). Between different cell types of skin, keratinocytes had a much higher expression of PAR2 than lymphatic endothelial cells and fibroblasts (Figure 3G). To determine whether keratinocyte rather than inflammatory cell/haematopoietic cell expression of PAR2 is necessary for lymphedema development, we created chimeric mice using bone marrow transplantation (BMT) from PAR2KO mice to WT mice (i.e., normal keratinocyte PAR2 expression and decreased expression by inflammatory/haematopoietic cells). Two months after BMT, over 80% of haematopoietic cells in blood samples from both groups expressed CD45.1, indicating successful transplantation (Figure S4A). In addition, to enable cell‐specific deletion, we created keratinocyte‐specific PAR2 conditional knockout mice by crossing KRT14Cre and PAR2 floxed mice (KRT14Cre^ERT2^/PAR2^fl/fl^; hereafter referred to as PAR2^cko^ for simplicity).

Three weeks after tail skin/lymphatic excision surgery in the bone marrow chimeric mice, we found that WT mice transplanted with bone marrow from WT mice (WT→WT) had tail swelling and lymphedema that was indistinguishable from WT controls, suggesting that inflammatory cell expression of PAR2 is not needed for lymphedema development. In contrast, PAR2KO mice transplanted with WT bone marrow (WT→PAR2KO) had decreased swelling (Figures 3H and S4B). These findings were supported by our observation of decreased tail swelling in PAR2^cko^ mice when PAR2 was conditionally knocked out in keratinocytes (Figures 3I and S4C).

We also noted that there was no significant difference among the relative changes in tail volume of PAR2KO, WT→PAR2KO and PAR2^cko^ mice compared with their respective controls (Figure 3J). Both WT→PAR2KO and PAR2^cko^ mice had decreased epidermal thickness and expression of PAR2, TSLP and IL‐33 (Figures 3K–N and S4D,E). Loss of PAR2 expression in keratinocytes decreased the number of Th2 cells in the skin and draining lymph nodes. Although the number of LCs was also lower in the draining lymph nodes, we noted a similar expansion of these cells in the skin of WT→PAR2KO and PAR2^cko^ mice but not in WT→PAR2KO and PAR2^cko^ mice (Figures 3O,P and S4F,G). Importantly, similar to the global PAR2KO mice, WT→PAR2KO and PAR2^cko^ mice had markedly decreased pathological changes of lymphedema, including skin changes, inflammation and fibrosis, suggesting that PAR2 expression in keratinocytes plays a more important role in the pathophysiology of secondary lymphedema than the expression of this molecule by other cells.

### 2.4 Topical inhibition of TSLP decreases the pathology of secondary lymphedema

**FIGURE 4:**
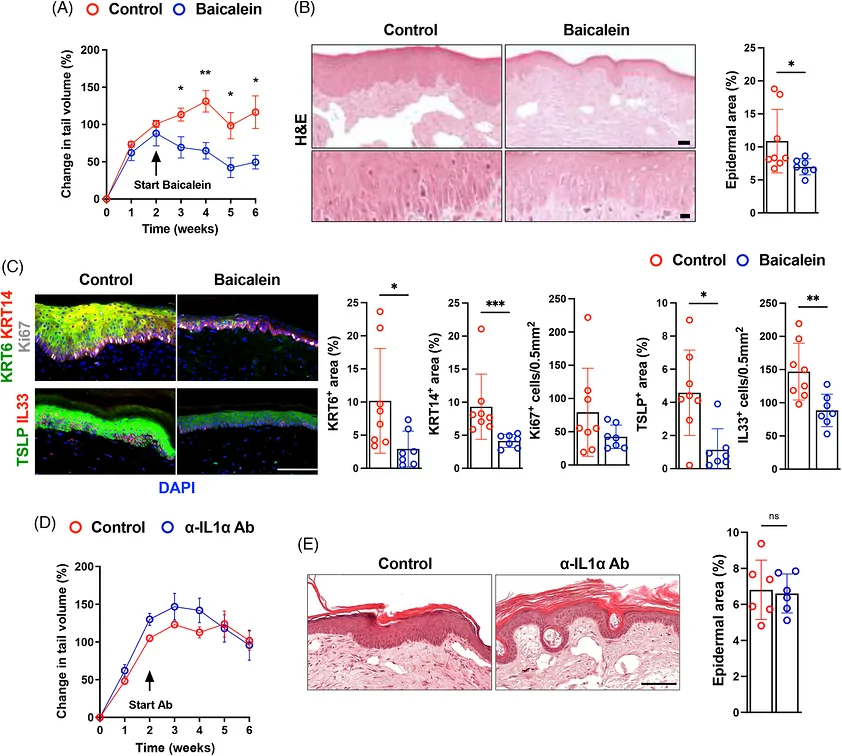
Inhibition of TSLP reduces lymphedema pathology. (A) Changes in tail volume over time in mice treated with vehicle (control) or baicalein once daily for 4 week beginning 2 week after tail skin and lymphatic excision. Tail skin was harvested 6 week after skin and lymphatic excision. Each circle represents the average measurement from each mouse (*n *= 7–8). *p < .05, **p < .01. *p* Values were calculated by two‐way ANOVA. (B) Representative H&E images and quantification of the epidermal area in the tail skin from mice treated with vehicle (control) or baicalein. Each circle represents the average of quantification of three HPF views for each mouse (*n *= 7–8). Scale bars: low magnification (top), 50 µm; high magnification (bottom), 10 µm. *p < .05. *p* Values were calculated by Mann–Whitney test. (C) Representative immunofluorescent images (left) and quantification (right) of KRT6, KRT14, Ki67, TSLP and IL‐33 staining in the tail skin from mice treated with vehicle (control) or baicalein. Scale bar: 100 µm. Each circle represents the average quantification of three HPF views for each mouse (*n *= 7–8). *p < .05, **p < .01, ***p < .001. *p* Values were calculated by Mann–Whitney test. (D) Changes in tail volume over time in mice treated with isotype (control) or IL‐1α–neutralising antibody twice a week for 4 week starting 2 week after tail skin and lymphatic excision. Tail skin was harvested 6 week after tail skin and lymphatic excision. Each circle represents the average measurement from each mouse (*n *= 6). (E) Representative H&E images and quantification of the epidermal area in the tail skin from mice treated with isotype (control) or IL‐1α–neutralising antibody. Each circle represents the average quantification of three HPF views for each mouse (*n *= 6). Scale bar: 100 µm. *p* Values were calculated by Mann–Whitney test.

Keratinocyte expression of PAR2 plays a key role in the pathology of AD, another Th2‐dependent disease, by increasing the expression of Th2‐inducing cytokines such as TSLP.^39^, ^45^ Therefore, we next sought to determine whether inhibiting TSLP expression, similar to PAR2 inhibition, can prevent lymphedema development. To do this, we used a topical formulation of 2 mM baicalein, a small‐molecule inhibitor of TSLP, applied once daily for 4 week to the mouse tails beginning 2 week after skin/lymphatic excision.^8^, ^40^, ^46^, ^47^ Control mice were treated topically with vehicle only (Aquaphor^®^ ointment). Beginning 1 week after its initiation, treatment with baicalein significantly decreased tail lymphedema (Figure 4A). Histological analysis 6 week after tail/skin excision demonstrated significant reductions in epidermal thickness, proliferation (Ki67^+^) and expression of KRT6, KRT14 and Th2‐inducing cytokines (Figure 4B,C). Baicalein treatment restored the columnar orientation of the basal layer compared with the disoriented irregular shape in the controls (Figure 4B). H&E staining also revealed decreased tissue thickening compared with vehicle‐treated controls (Figure S6A). Furthermore, immunofluorescence analysis revealed less dilated LYVE1^+^ lymphatic structures in baicalein‐treated mice, indicating improved lymphatic drainage (Figure S6B). To confirm that these improvements in lymphedema were related to PAR2KO or baicalein treatment rather than to a nonspecific anti‐inflammatory effect, we treated other mice with monoclonal anti‐IL‐1α antibodies administered intraperitoneally (once a week, 5 µg/g) for 4 week beginning 2 week after tail skin/lymphatic excision surgery. Control mice were treated with isotype control antibodies. In contrast to the loss of PAR2 expression or baicalein treatment, this treatment had no effect on tail swelling or epidermal changes (Figure 4D,E).

### 2.5 LF activates keratinocyte proliferation and cytokine expression by a PAR2‐dependent mechanism

**FIGURE 5:**
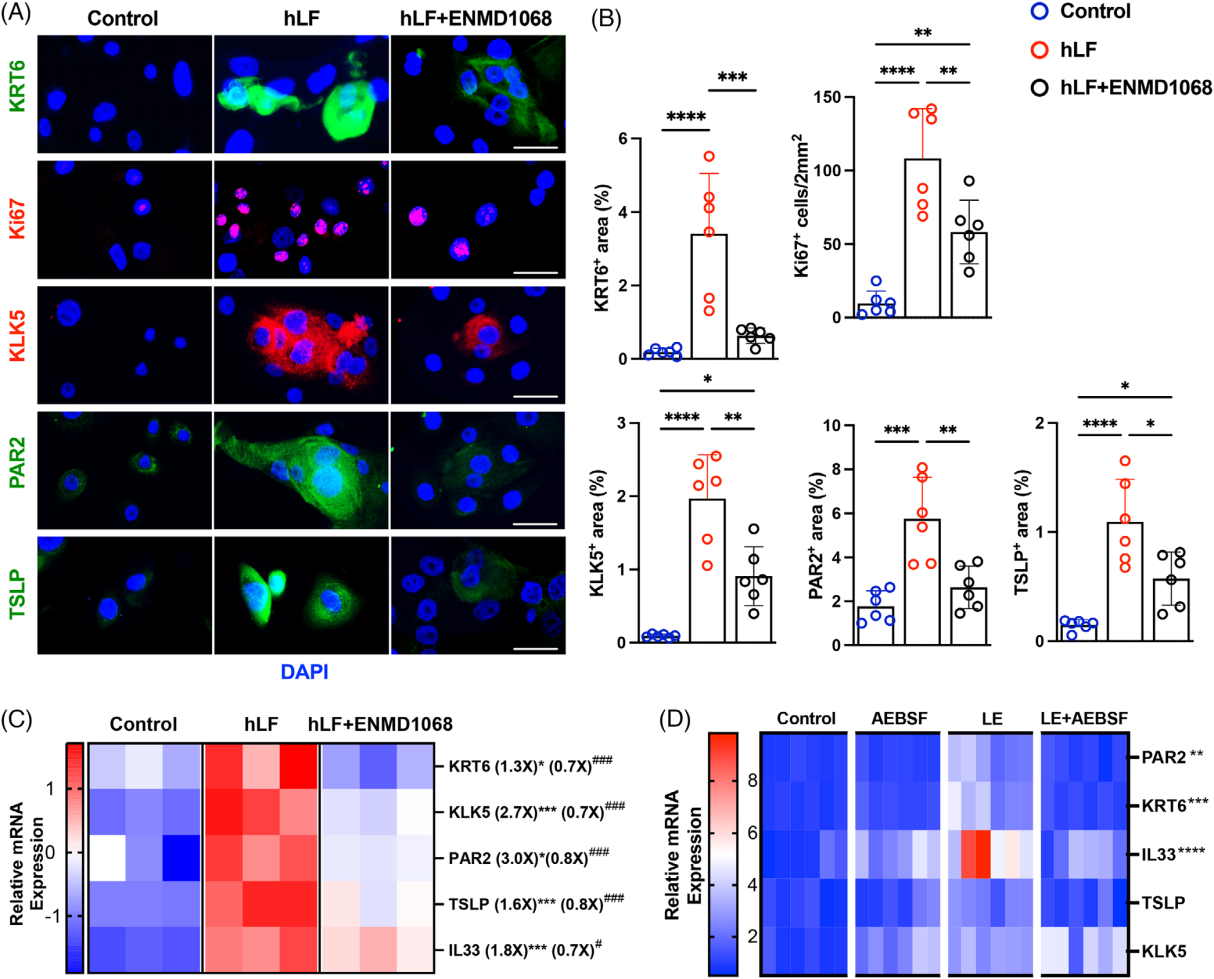
LF induces proliferation and increases the expression of PAR2 and Th2‐inducing cytokines in h‐keratinocytes. (A) Representative immunofluorescent images of KRT6, Ki67, KLK5, PAR2 and TSLP staining in h‐keratinocytes cultured with PBS (control), hLF or hLF+ENMD1068. Scale bar: 50 µm. (B) Quantification of the KRT6, Ki67, KLK5, PAR2 and TSLP areas in h‐keratinocytes cultured with PBS (control), hLF or hLF+ENMD1068. Each circle represents the average quantification of two HPF views for each experiment. LF from two different lymphedema patients was used (*n *= 6; 3 for each LF). *p < .05, **p < .01, ***p < .001, ****p < .0001. *p* Values were calculated by one‐way ANOVA. (C) Relative mRNA expression by qPCR in cultured h‐keratinocytes treated with PBS (control), hLF or hLF+ENMD (*n *= 3). mRNA expression was normalised by β‐actin expression. Each box represents each experiment with independently cultured h‐keratinocytes. Fold changes are relative to control for LF and relative to LF for LF+ENMD. *p indicates LF compared with control, and #*p* indicates LF+ENMD compared with LF. *p < .05, #*p* < .05, ***p < .001, ###*p* < .001. *p* Values were calculated by Mann–Whitney test. (D) Relative mRNA expression by qPCR in cultured h‐keratinocytes treated with PBS (control), AEBSF, hLF or hLF+AEBSF (*n *= 6). mRNA expression was normalised by β‐actin expression. Each box represents each experiment with independently cultured h‐keratinocytes. Fold changes are relative to control for AEBSF, LF and LF+AEBSF. *p indicates LF compared with LF+AEBSF. *p < .05, #*p* < .05, ***p < .001. *p* Values were calculated by two‐way ANOVA.

Because lymphatic injury results in the accumulation of interstitial fluid in the skin,^48^ we next sought to determine if LF can activate keratinocytes and regulate the expression of PAR2/Th2‐inducing cytokines. We collected LF from the affected arms of patients with secondary lymphedema and cultured human keratinocytes (h‐keratinocytes) with or without human LF (hLF). Because harvesting interstitial fluid from normal tissues in the volumes needed for our experimental studies is not possible, control cells were treated with media alone. Culturing h‐keratinocytes with hLF significantly increased the protein and mRNA expression of KRT6, Ki67, KLK5, PAR2 and TSLP 48 h after the treatment (Figure 5A–C). The addition of a small‐molecule inhibitor of PAR2 (ENMD1068) decreased the expression of KRT6 and PAR2 to the same levels as the controls and significantly decreased the expression of Ki67, KLK5 and TSLP (Figure 5A–C). To further investigate whether protease activity within LF contributes to this effect, we co‐treated keratinocytes with hLF and the broad‐spectrum serine protease inhibitor 4‐(2‐aminoethyl) benzene sulfonyl fluoride hydrochloride (AEBSF).^49^ This intervention led to a significant reduction in PAR2, KRT6 and IL33 expression (Figure 5D), supporting a protease‐dependent mechanism underlying LF‐induced keratinocyte activation.

### 2.6 LF contains proteases and induces TSLP expression in keratinocytes in a PAR2‐dependent fashion

**FIGURE 6:**
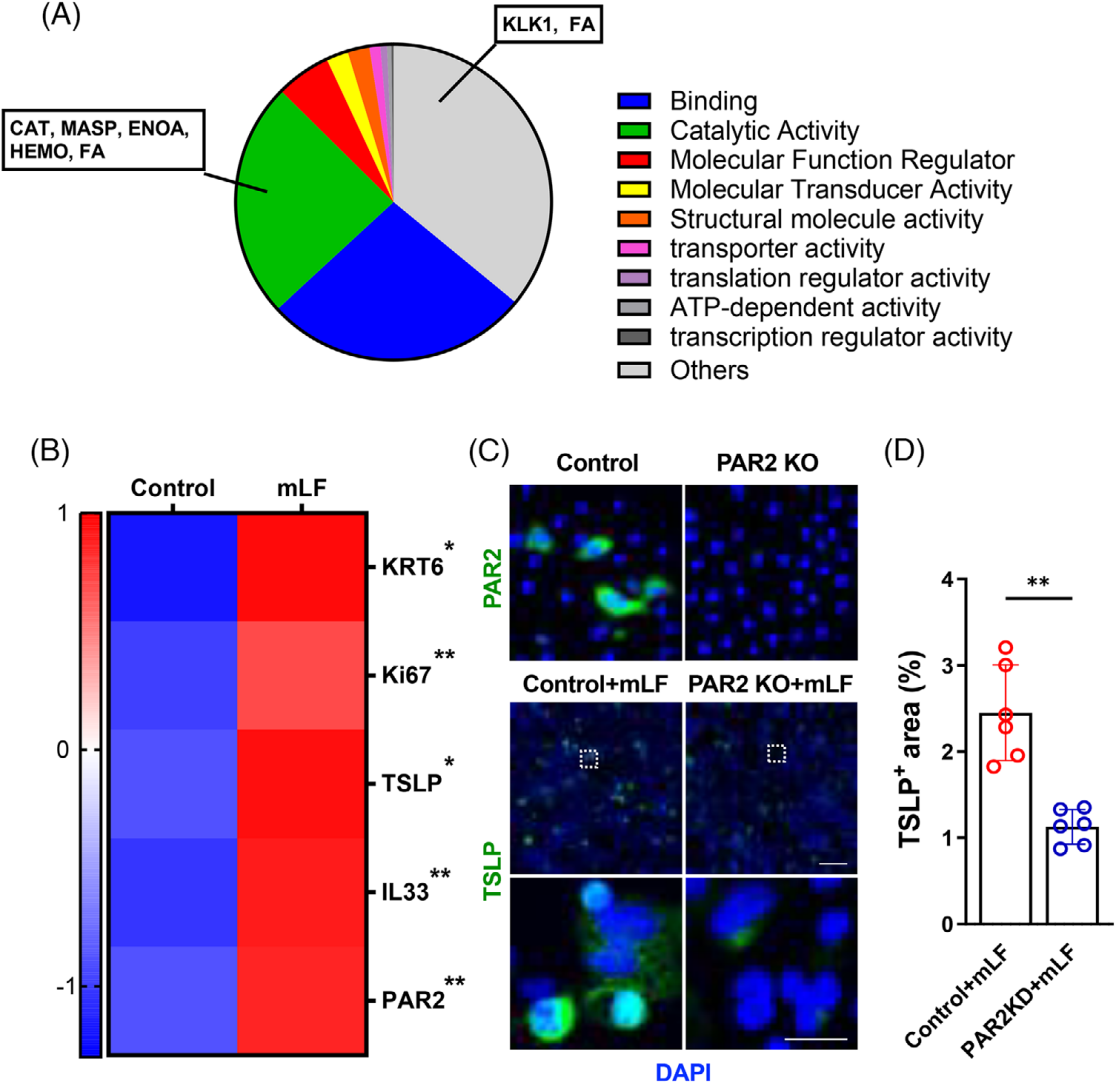
LF induces TSLP expression of m‐keratinocytes in a PAR2‐dependent fashion. (A) Proteomic analysis of mLF identified 351 proteins, of which 108 appeared to have catalytic activity. (B) Relative mRNA expression by qPCR in m‐keratinocytes cultured with PBS (control) or mLF (*n *= 3). mRNA expression was normalised to β‐actin expression. Each box represents each experiment with independently cultured m‐keratinocytes. *p < .05, **p < .01. *p* Values were calculated by *t*‐test. (C) Representative immunofluorescent images of PAR2 staining in m‐keratinocytes treated with control siRNA or PAR2 knockdown siRNA. Representative immunofluorescent images of TSLP staining in m‐keratinocytes cultured with PBS (control) and mLF after PAR2 knockdown. Scale bar: 50 µm. (D) Quantification of the TSLP area in m‐keratinocytes cultured with PBS (control) or mLF after PAR2 knockdown. Each circle represents the average quantification of two HPF views for each experiment (*n *= 6). ***p < .001. *p* Values were calculated by one‐way ANOVA.

To determine which components of LF activate keratinocytes, we harvested LF from the tails of mice (mouse LF [mLF]) 2 week after tail skin/lymphatic excision surgery. Proteomic analysis identified 351 proteins, which we categorised by their molecular function. Interestingly, 108 of the 351 proteins appeared to have catalytic activity, and we found several serine proteases that activate PAR2, including cathepsin, coagulation factors, enolase, KLK1 and MT‐SP (Figure 6A).^50^, ^51^, ^52^, ^53^, ^54^ We cultured primary mouse keratinocytes (m‐keratinocytes) with mLF and found that mLF increased expression of KRT6, Ki67, TSLP, IL‐33 and PAR2 (Figure 6B). Consistent with our findings using h‐keratinocytes, we found that PAR2 knockdown in m‐keratinocytes treated with mLF also markedly decreased TSLP expression compared with the controls (Figure 6C,D).

### 2.7 Teriflunomide treatment inhibits hLF‐induced keratinocyte proliferation in vitro

**FIGURE 7:**
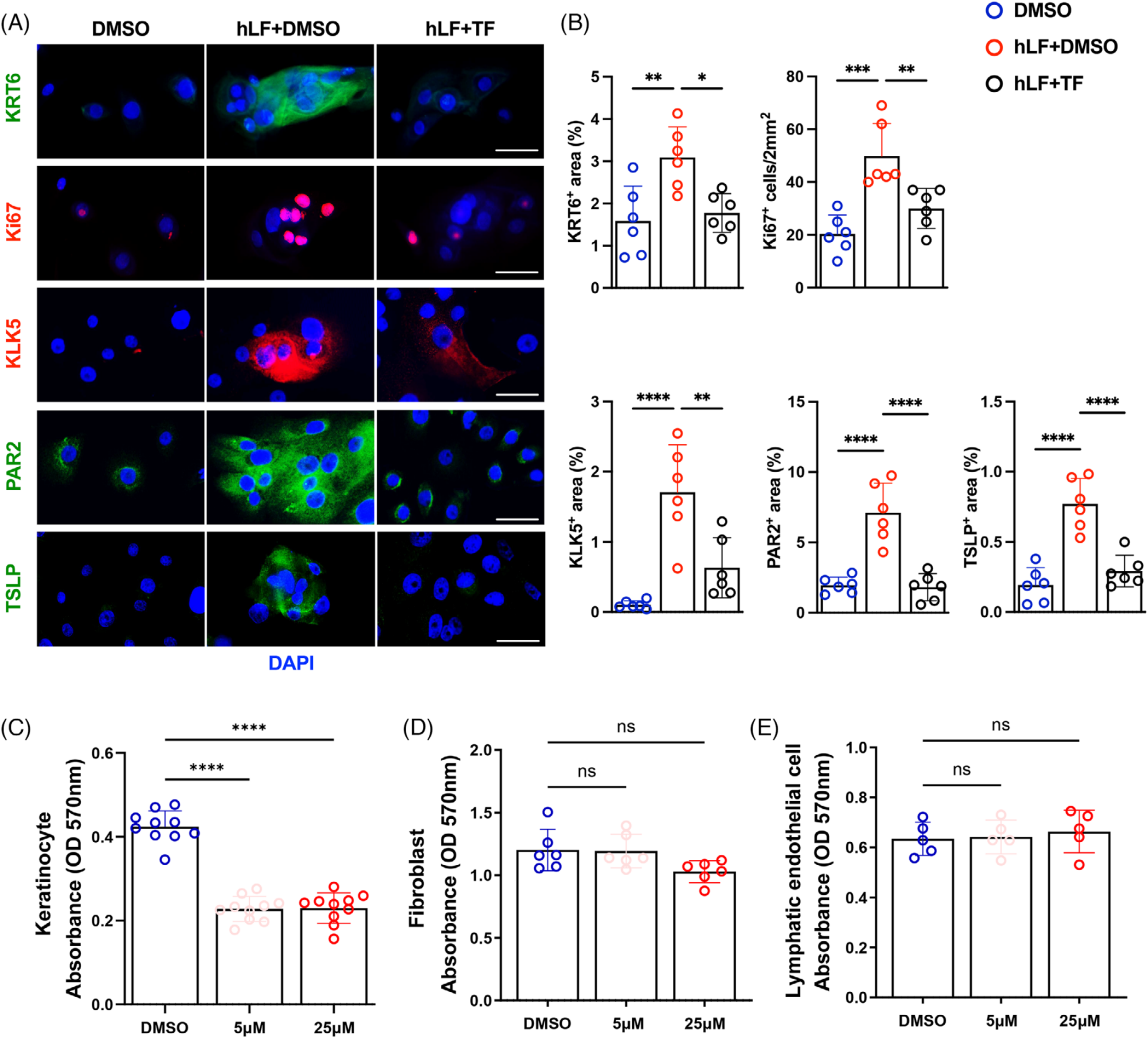
TF treatment inhibits hLF‐induced keratinocyte proliferation in vitro. (A) Representative immunofluorescent images of KRT6, Ki67, KLK5, PAR2 and TSLP staining of h‐keratinocytes cultured with DMSO, hLF+DMSO or hLF+TF (25 µM in DMSO). Scale bar: 50 µm. (B) Quantification of the KRT6, Ki67, KLK5, PAR2 and TSLP areas in h‐keratinocytes cultured with DMSO, hLF+DMSO or hLF+TF. Each circle represents the average quantification of two HPF views for each experiment (*n *= 6). *p < .05, **p < .01, ***p < .001, ****p < .0001. *p* Values were calculated by one‐way ANOVA. Proliferation (MTT assay) of (C) h‐keratinocytes, (D) fibroblasts and (E) lymphatic endothelial cells cultured with DMSO only or TF (*n *= 10). Each circle represents an individual experiment. ****p < .0001. *p* Values were calculated by one‐way ANOVA.

A major effect of lymphatic injury in vivo or of culturing keratinocytes with hLF in vitro was rapid and sustained proliferation of keratinocytes in the basal layer of the epidermis. Therefore, we hypothesised that keratinocyte proliferation may also play a role in the regulation of PAR2 and Th2‐inducing cytokine expression.^55^, ^56^, ^57^ To test this hypothesis, we first treated human keratinocytes with teriflunomide (TF) in vitro. TF is the active metabolite of leflunomide and inhibits de novo synthesis of pyrimidine by blocking dihydroorotate dehydrogenase and inhibiting cellular proliferation. It is currently a United States Food and Drug Administration (US FDA)‐approved treatment for multiple sclerosis.^58^, ^59^ Treatment with 25 µM TF significantly abrogated h‐keratinocyte proliferation in vitro in response to exposure to hLF. TF also significantly decreased the expression of KRT6, Ki67, KLK5, PAR2 and TSLP in LF‐stimulated h‐keratinocytes (Figure 7A,B). In fact, TF was even more effective than ENDM1068 in decreasing TSLP expression to baseline levels. In contrast, TF treatment or exposure to hLF had no effect on other skin cells such as fibroblasts or lymphatic endothelial cells (Figure 7C–E).

### 2.8 TF decreases epidermal changes and other pathological changes of secondary lymphedema

**FIGURE 8:**
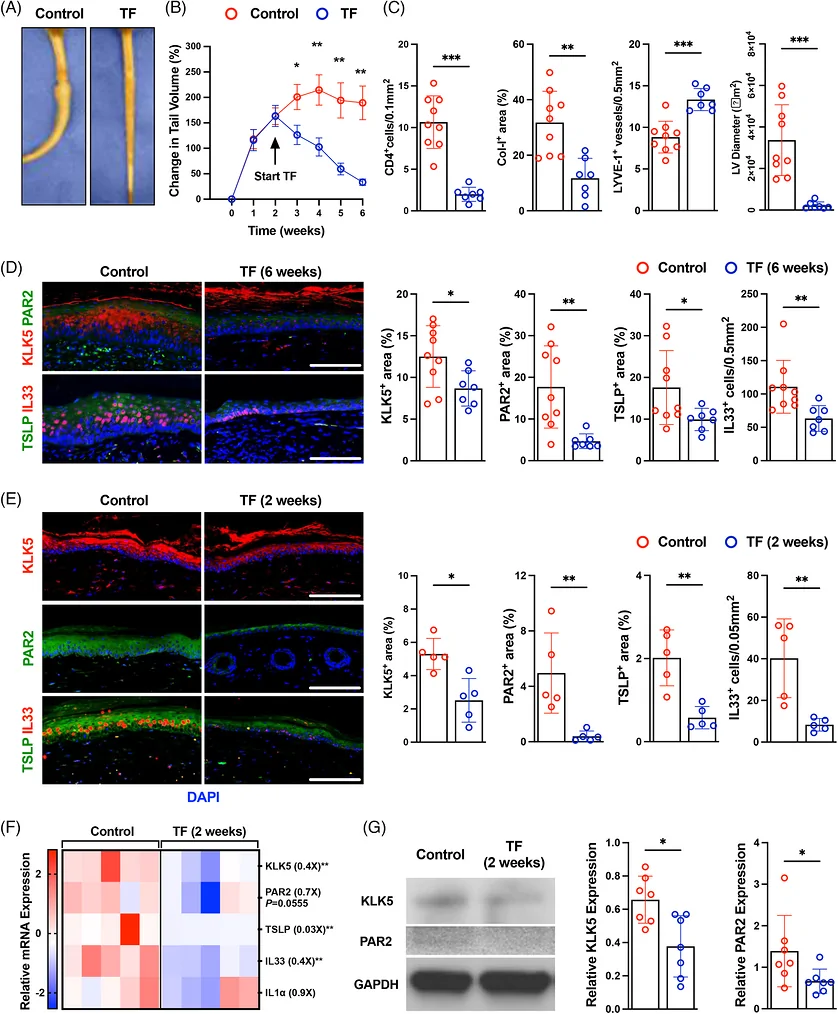
Topical treatment with TF prevents lymphedema development. (A) Representative images of control and TF‐treated mice 6 week after tail skin and lymphatic excision. (B) Changes in tail volume over time in mice treated with vehicle (control) or TF once daily for 4 week starting 2 week after tail skin and lymphatic excision. Tail skin was harvested 6 week after tail skin and lymphatic excision. Each circle represents the average measurement from each mouse (*n *= 7–9). *p < .05, **p < .01. *p* Values were calculated by two‐way ANOVA. (C) Quantification of CD4^+^ cells, collagen I positivity, LYVE1^+^ vessels and lymphatic vessel diameter in skin samples harvested from mice treated with vehicle (control) or TF. Each circle represents the average quantification of three HPF views for each mouse (*n *= 7–9). **p < .01, ***p < .001. *p* Values were calculated by Mann–Whitney test. (D) Representative immunofluorescent images (left) and quantification (right) of KLK5, PAR2, TSLP and IL‐33 staining in the tails of mice treated with vehicle (control) or TF. Scale bar: 100 µm. Each circle represents the average quantification of three HPF views for each mouse (*n *= 5). *p < .05, **p < .01. *p* Values were calculated by Mann–Whitney test. (E) Representative immunofluorescent images (left) and quantification (right) of KLK5, PAR2, TSLP and IL‐33 staining in the tails of mice treated with vehicle (control) or TF once daily for 2 week starting 1 day after tail skin and lymphatic excision. Tail skin is harvested 2 week after tail skin and lymphatic excision. Scale bar: 100 µm. Each circle represents the average quantification of three HPF views for each mouse (*n *= 5). *p < .05, **p < .01. *p* Values were calculated by Mann–Whitney test. (F) Relative mRNA expression by qPCR in the tail skin of mice treated with vehicle (control) or TF (*n *= 5). mRNA expression was normalised to β‐actin expression. Each box represents one mouse. **p < .01. *p* Values were calculated by Mann–Whitney test. Fold change from control is shown in parentheses. (G) Representative images of western blots (left) and quantification (right; relative to GAPDH) of KLK5 and PAR2 in the tail skin of mice treated with vehicle (control) or TF. Each dot represents quantification of a separate western blot (*n *= 7). *p < .05. *p* Values were calculated by Mann–Whitney test.

Because TF was highly effective in inhibiting the proliferative response of keratinocytes to hLF, we next tested the hypothesis that topical TF is also effective as a treatment for lymphedema in the mouse tail model. To test this, we treated mice once a day for 4 week with 27 mg/mL of topical TF or vehicle control (Aquaphor^®^) beginning 2 week after tail skin/lymphatic excision surgery. This treatment decreased tail swelling almost immediately, with significant changes noted after 1 week and all remaining time points (Figure 8A,B). At the conclusion of the experiment, TF‐treated mice had virtually no swelling.

Consistent with improved lymphedema outcomes, we also found that mice treated with topical TF had decreased dermal infiltration of CD4^+^ cells, decreased type I collagen deposition, an increased number of lymphatic capillaries, a decreased lymphatic vessel diameter and decreased expression of KLK5, PAR2, TSLP and IL‐33 compared with control mice (Figures 8C,D and S5A). To investigate whether TF has a dose‐dependent effect on lymphedema, we treated mice with a low dose of topical TF (14 mg/mL) once a day for 4 week after tail skin/lymphatic excision surgery. Low‐dose topical TF significantly reduced tail swelling after 4 week of treatment, whereas high‐dose topical TF reduced tail swelling within 1 week of treatment, showing that topical TF has a dose‐dependent effect on lymphedema (Figure S5B).

To investigate whether treatment with topical TF can mitigate early pathological changes in keratinocytes after lymphatic injury, we repeated the experiment, beginning TF treatment immediately after surgery and dosing once daily for 2 week. This approach reduced tail swelling and markedly decreased skin expression of KLK5, PAR2, TSLP, IL‐13, IL‐33, Ki67, KRT6, IL‐1α and NFATc1 (Figures 8E–G and S5C–E). Our analysis of the number of proliferating cells in the dermis showed no difference before and after TF treatment, confirming that the effects of TF were primarily on keratinocytes in the epidermis rather than on other cell types in the dermis (Figure S5F).

## 3 DISCUSSION

The skin is the largest organ in the body and consists of the epidermis, dermis and hypodermis. Keratinocytes are skin cells that make up 90% of the epidermis and originate as stem cells in basal layers of the skin. Keratinocytes proliferate, differentiate and migrate to the more superficial layers of the skin, ultimately forming the cornified layer. Major functions of keratinocytes include maintenance of skin barrier function, prevention of water loss and inhibition of bacterial infiltration.^60^ Proliferation and differentiation of keratinocytes is controlled by diverse cytokines such as IL‐1α, IL‐1β, EGF, TGF1α and TNFα.^61^ Keratins (KRT) are a large family of intermediate filaments that are expressed by keratinocytes and are necessary for maintenance of cytoskeletal integrity and cellular motility.^35^ Keratins KRT14–KRT5 are expressed by keratinocytes and keratinocyte precursors in the stratum basale. As the cells migrate suprabasally and become differentiated, expression of KRT14–KRT5 heterodimers is replaced by KRT10–KRT1. KRT16, KRT17 and KRT6 are expressed in activated, proliferating keratinocytes in pathological or physiological conditions such as AD, psoriasis, wound healing and burns.

**FIGURE 9:**
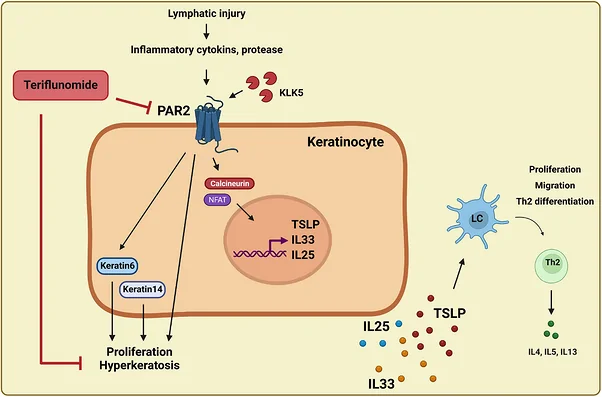
Model of keratinocyte‐regulated epidermal changes in lymphedema. In the early stages of secondary lymphedema, lymphatic injury induces LF accumulation and activates hyperkeratosis accompanied by proliferation and increased KRT6 and KRT14 expression. The expression of KLK5–PAR2 and Th2‐inducing cytokines (TSLP, IL‐33, IL‐25) is up‐regulated in the keratinocytes of lymphedematous skin, resulting in LC activation and Th2 differentiation. Topical application of TF inhibits hyperkeratosis, as well as PAR2‐induced Th2‐inducing cytokine expression, eventually reducing the pathophysiology of secondary lymphedema.

Hyperkeratosis is a histological hallmark of lymphedema and a common finding in inflammatory skin disorders.^23^, ^24^ Although keratinocytes are known to play a key role in the pathophysiology of psoriasis and AD, no prior studies have tested the hypothesis that these cells also contribute to the pathology of secondary lymphedema.^28^, ^62^ In this study, we used clinical samples collected from women with unilateral BCRL to show that lymphedema increases proliferation and decreases differentiation of keratinocytes in the basal layer of the skin. Keratinocytes in lymphedematous skin are activated as evidenced by increased expression of KRT6, KRT16 and KRT17. Using mouse models of lymphedema, we found that epidermal changes occur rapidly after lymphatic injury and that these changes precede infiltration of CD4^+^ cells. The surgical model of lymphatic injury result in rapid pathological epidermal changes, suggesting that this effect is independent of wound healing. LF activated h‐keratinocyte proliferation and expression of KRT6 in vitro support this hypothesis and suggest that stagnant LF accumulation due to increased lymphatic permeability and decreased lymphatic clearance is an important regulator of keratinocyte changes. These findings are consistent with a previous report demonstrating that LF induced keratinocyte proliferation and expression of KRT6, KRT16 and KRT17, and that this response was mitigated by inhibiting IL‐1β, keratinocyte growth factor or TNF‐α.^23^ Taken together, our data suggest that lymphatic injury rapidly activates keratinocytes to induce hyperkeratosis in the early stages of lymphedema, and this is accompanied by proliferation, inhibited differentiation and increased expression of activation markers, such as KRT6 (Figure 9).

Lymph is fluid that accumulates in interstitial space and is transported by lymphatic vessels. The composition of LF is regulated by physiologic factors as well as by inflammatory responses elicited by trauma, haemorrhagic shock or lymphatic injury.^63^ Tissue injury causes an imbalance in the ratio of protease inhibitor to protease in lymph, resulting in a net activation of protease activity and induction of inflammatory responses.^64^ Consistent with these observations, we found that the expression of PAR2, a protease receptor, and KLK5, an endogenous skin protease, is significantly increased after lymphatic injury and in lymphedema skin biopsies from patients with unilateral BCRL. PAR2 is a transmembrane protein that is expressed primarily in the skin and activated by proteases including KLK5, trypsin and papain.^65^ KLK5 is the most important skin protease that promotes barrier dysfunction in inflammatory skin diseases.^33^, ^41^

We found that PAR2 inhibition with ENMD1068, a small‐molecule inhibitor, decreased the activation of keratinocytes by LF in vitro and decreased the expression of Th2‐inducing cytokines, such as TSLP and IL‐33. More importantly, keratinocyte‐specific inhibition of PAR2 activation in transgenic mice significantly decreased the pathological findings of lymphedema (i.e., fibrosis, swelling) and decreased Th2 inflammatory responses. Loss of PAR2 signalling after skin/lymphatic excision decreased the number of activated LCs in regional lymph nodes and the number of infiltrating Th2 cells in the tail skin and draining lymph nodes. Our findings are consistent with previous studies demonstrating that overexpression of KLK5 results in the development of Netherton syndrome, inflammatory skin lesions and increased Th2 cell infiltration.^66^ In contrast, KLK5 inhibition reverses the pathology of Netherton syndrome.^33^ Similarly, PAR2 overexpression in basal keratinocytes increases allergic responses to house dust mites and skin inflammation in mouse models of AD.^67^ In contrast, PAR2 inactivation reduces early production of TSLP, decreases inflammation and ichthyosis and decreases Th2 inflammation in mouse models of AD and Netherton syndrome.^41^, ^68^, ^69^

Several lines of evidence suggest that PAR2 regulates proliferation, differentiation and activation of inflammatory gene expression in keratinocytes, whereas others suggest it plays this role in inflammatory cells and endothelial cells.^43^, ^44^, ^70^, ^71^ Our single‐cell RNAseq data showed that PAR2 was dominantly enriched in keratinocyte clusters in skin biopsies from patients with unilateral BCRL. However, analysis across three patients revealed no consistent increase in the proportion of F2RL1/PAR2‐positive keratinocytes, underscoring interpatient variability and potential intra‐population heterogeneity, as observed in our bulk RNA‐seq analysis. This apparent discrepancy with protein‐level analyses may be explained by state‐specific changes revealed by single‐cell analysis and by a relatively small sample size. Specifically, LE skin exhibited a marked shift in keratinocyte state composition, with expansion of differentiating and mesenchymal‐like keratinocyte states and reduction of basal populations. Within these disease‐enriched states – particularly differentiating and immune‐interacting keratinocytes – there was a pronounced increase in the fraction of F2RL1/PAR2‐expressing cells, whereas proliferative keratinocytes showed reduced expression.

Together, these findings indicate that increased PAR2 signalling in LE is not uniform across all keratinocytes but is instead driven by both cell‐state redistribution and state‐specific regulation of F2RL1/PAR2. This may resolve the discrepancy between single‐cell analyses and protein‐level analyses and highlight that keratinocytes actively participate in the inflammatory microenvironment of lymphedematous skin. Specifically, differential enrichment of F2RL1 expression in differentiating, mesenchymal‐like and immune‐associated keratinocyte states suggests that PAR2 signalling is a central pathway linking keratinocyte activation to Th2 inflammation, altered differentiation, epithelial–mesenchymal transition–like changes^72^ and epidermal barrier dysfunction in secondary lymphedema.^73^

These observations raised the possibility that keratinocyte‐intrinsic PAR2 signalling is not merely associated with, but functionally drives the pathological epidermal remodelling observed in lymphedema. Using three different mouse models (PAR2‐deficient mice, chimeric mice with BMT and keratinocyte‐specific conditional PAR2KO mice), we found that lymphedema phenotypes and skin changes were reduced in all three; there was no significant difference among the groups, suggesting that rather than expression by other cell types, keratinocyte expression of PAR2 is critical for lymphedema development.

We found that, similar to AD and other inflammatory skin disorders, keratinocytes in human and mouse lymphedema samples express Th2‐inducing cytokines (TSLP, IL‐33 and IL‐25). These findings are important because TSLP, IL‐33 and IL‐25 are epithelium‐derived cytokines that regulate Th2 inflammation in a variety of settings, including AD, allergic rhinitis, psoriasis, food allergy and allergic asthma.^31^, ^74^ Overexpression of TSLP in mouse skin leads to spontaneous AD and increased Th2 cell responses.^75^ Topical treatment with vitamin D3 analogue MC903 induces an AD‐like phenotype and TSLP overexpression in keratinocytes and an increased allergic reaction in an allergic asthma mouse model, whereas a KRT14‐specific TSLP mutation reverses the effect of MC903.^74^ Deficiency of TSLP receptor in CD11c^+^ DCs reduces Th2 cell responses in a mouse model of lung inflammation, suggesting that DCs respond to epithelial‐derived TSLP.^76^ Indeed, TSLP stimulates DC activation by inducing costimulatory molecules, such as OX40L, CD80 and CD86, and TSLP‐stimulated DCs promote Th2 polarisation.^31^ Innate immune cells (ILCs) and granulocytes (eosinophil, basophil, mast cells) respond to TSLP and produce inflammatory Th2 cytokines, such as IL‐4 and IL‐13.^31^ IL‐33 and IL‐25 also induce Th2 immune responses by acting directly on CD4^+^ T cells, Th2 memory cells and ILC2s.^32^ Taken together, substantial evidence suggests that epithelial‐derived Th2‐inducing cytokines regulate Th2 differentiation and inflammatory disorders, and that these responses may also drive inflammatory responses in lymphedema.

The interaction between keratinocytes and Th2 inflammatory cytokines is bidirectional. Keratinocyte‐derived Th2‐inducing cytokines drive Th2 differentiation; in turn, Th2 cytokines, such as IL‐4 and IL‐13, regulate keratinocyte differentiation and barrier function.^77^ In vitro treatment of skin‐equivalent models with Th2 cytokines results in disturbed keratinocyte differentiation and an AD‐like skin phenotype.^78^ Th2 cytokines also inhibit the expression of skin barrier proteins, such as filaggrin, loricrin and involucrin, leading to increased skin permeability and sensitivity to bacterial toxins.^79^ Th2 cytokines also regulate keratinocyte expression of Th2‐inducing cytokines, thus acting in a feed‐forward manner.^80^ Our recent clinical trial testing the safety of monoclonal anti‐IL‐4/IL‐13 antibody treatments in patients with unilateral BCRL is consistent with this paradigm. We observed decreased expression of keratinocyte‐derived Th2‐inducing cytokines and immune cell recruitment after a 4‐months treatment course.^20^ Changes in skin barrier function related to Th2 cytokine expression may also serve as a putative mechanism for the increased risk of skin infections in some patients with lymphedema and recent reports demonstrating clinical evidence of skin barrier dysfunction and increased transepidermal water loss.^81^ Inhibiting T cells and neutralising IL‐4/IL‐13 the downstream targets of lymphedema pathology might be complemented with inhibiting the upstream PAR2–TSLP pathway might have a synergistic effect in treating lymphedema.

Our finding of abnormal keratinocyte proliferation in lymphedema led us to hypothesise that treatment with TF, an US FDA‐approved proliferation inhibitor, would be an effective treatment for this disease.^82^ The drug's mechanism of action is inhibition of cellular proliferation and induction of apoptosis of undifferentiated cells.^83^, ^84^ Indeed, we found that in vitro treatment of LF‐stimulated keratinocytes with TF markedly decreased cellular proliferation and expression of PAR2 and Th2‐inducing cytokines. These findings are consistent with previous reports demonstrating that inhibition of PAR2 decreases keratinocyte proliferation, suggesting that PAR2 expression activates a positive feedback response with cellular proliferation.^70^ It is possible that topical TF used in our mouse models also directly decreases inflammatory responses, as TF can also inhibit proliferation of inflammatory cells. However, the finding that treatment with TF shortly after lymphatic injury (i.e., before infiltration of inflammatory cells) also decreased hyperkeratosis and expression of PAR2 suggests that the beneficial effects may be due primarily to the effect of TF on keratinocytes.

Based on these preclinical findings, in future, TF can be a potential drug candidate to treat lymphedema in humans using topical formulations. However, its safety and efficacy of TF as a topical cream for lymphedema should be thoroughly tested. It will be important to understand how much TF will get into the system and how it is affecting the inflammatory cells systemically for the safety concern.

## 4 LIMITATIONS

Our study has some limitations. Although our mouse lymphedema models closely correlate with the histological and inflammatory changes in BCRL, it is possible that these models do not completely reflect the clinical scenario. However, it is important to note that there are currently no reproducible large animal models of lymphedema. Canine models require long‐term (>6 months) follow‐up after surgery and result in lymphedema development in only a subset of animals^85^; pig and sheep models are more consistent with simple lymphatic injury because the procedures do not cause significant, sustained swelling or fibrosis.^85^ LF was collected only from stage II lymphedema patients, as stage I lymphedema patients are rarely candidates for lymphedema surgery and generally do not have enough fluid in the skin at the time of surgery that we can collect for experimental purpose. However, we observed that some stage I patients exhibited increased epidermal area and elevated Th2‐inducing cytokine expression in the skin. This overlap in presentation leads to the conclusion that LF from stage II patients can serve as a proxy for or adequately represent the stage I condition. We only used LF from lymphedematous skin because there is no interstitial fluid in normal skin that can be harvested for large‐scale studies. In addition to the keratinocyte activation through PAR2, variety of non‐cell autonomous mechanisms including cytokines (IL‐33), interstitial fluid stasis, neuropeptides, proteases, extracellular matrix components and microbial factors can influence PAR2 expression on keratinocytes and regulate lymphedema pathology. Given the keratinocyte centric focus of this manuscript, these non‐cell autonomous mechanisms fall outside the scope of this study and would be ideal candidates for future studies.

## 5 CONCLUSIONS

In conclusion, our study indicates that keratinocytes play an active role in the development of lymphedema by coordinating Th2 inflammatory responses. Abrogation of these changes in keratinocytes is highly effective in preclinical models and may represent a novel approach for preventing or treating lymphedema.

## 6 MATERIALS AND METHODS

### 6.1 Patient samples

All procedures were approved by the Institutional Review Board (IRB protocol 17–377) at Memorial Sloan Kettering Cancer Center (MSK). Women with unilateral upper extremity BCRL were identified in our lymphedema clinic and screened for eligibility for harvesting of biopsy specimens. Inclusion criteria included age between 21 and 75 years, unilateral axillary surgery and stage I–III lymphedema (volume differential of >10% with the normal limb or L‐Dex measurements above 7.5 units). Exclusion criteria included pregnancy or lactating women, recent (within 3 months) history of lymphedematous limb infection, chemotherapy, treatment with steroids or other immunosuppressive agents and active cancer or breast cancer metastasis. We harvested excessive interstitial LF from the lymphedematous arm and 5‐mm full‐thickness skin biopsies from the volar surface of the normal and lymphedematous arms at a point located 5–10 cm below the elbow crease. Biopsy was performed under sterile conditions with local anaesthesia. Patients were treated with a dose of antibiotics (1000 mg cephalexin or 600 mg clindamycin for penicillin‐allergic patients) 30–60 min before the procedure. We obtained informed consent from all patients.

### 6.2 Animals

All studies were approved by the Institutional Animal Care and Use Committee (IACUC) at MSK (protocol 06‐08‐018). The MSK IACUC adheres to the National Institutes of Health Public Health Service Policy on Humane Care and Use of Laboratory Animals and operates in accordance with the Animal Welfare Act and the Health Research Extension Act of 1985. Per the IACUC‐approved protocol, all mice were maintained in light‐ and temperature‐controlled pathogen‐free environments and fed ad libitum.

Adult (8–12‐week‐old) female C57BL/6J mice were used for all treatment studies. We chose to use female mice for our study because secondary lymphedema affects females more commonly than males.^86^ PAR2KO, KRT14Cre and PAR2 floxed mice based on a C57BL/6J background were purchased from The Jackson Laboratory (B6.Cg‐F2rl1^tm1Mslb^/J, Tg(KRT14‐cre/ERT)20Efu/J and F2rl1tm1.1Tjp/J). For conditional knockouts, the expression of transgenes was confirmed by genotyping (Transnetyx, Cordova, TN), and double‐homozygous mice were backcrossed for six to seven generations to ensure consistency. For PAR2KO, age‐ and sex‐matched WT C57BL/6J control mice also purchased from The Jackson Laboratory. For conditional knockouts, age‐ and sex‐matched PAR2 floxed mice were used as controls.

### 6.3 Surgical model of lymphedema

Anaesthesia was induced using isoflurane (Henry Schein Animal Health), and mice were kept on a heating blanket to maintain body temperature. Depth of anaesthesia was monitored by reaction to pain and observation of respiratory rate. Animals were excluded from the experiment if wound infection or ulceration in the tail was noted at any time point after surgery. Postoperative pain control was maintained with three doses of intraperitoneal buprenorphine injection every 4–12 h. Animals were euthanised by carbon dioxide asphyxiation as recommended by the American Veterinary Medical Association.

In the tail surgery model, the superficial and deep lymphatic vasculatures were ligated through a 2‐mm circumferential excision of the skin 2 cm distal to the base of the tail. Collected lymphatics were identified using Evans blue injection and ligated along the entire length of the skin excision. Leakage of the of the blue dye at the site of injection is an indication of successful ligation of the vessels. Control animals underwent skin incision without lymphatic ligation.^15^, ^87^

### 6.4 Tail volume measurement

Tail volumes (*V*) were calculated weekly after tail surgery to evaluate the development of lymphedema over time.^88^ Digital calipers were used to measure tail diameter every 1 cm starting at the surgical site going distally towards the tip of the tail. Serial circumferences (*C*) were determined and used to calculate tail volume per the truncated cone formula [*V* = 1/4*π* (*C*_1_ *C*_2_ + *C*_2_ *C*_3_ + *C*_3_ *C*_4_)].

### 6.5 Histology and immunofluorescence

Histological and immunofluorescence analyses were performed using our published techniques.^40^ Clinical and experimental biopsy specimens were fixed in 4% paraformaldehyde (Sigma–Aldrich) overnight. Tails were decalcified using 5% EDTA (Santa Cruz), embedded in paraffin and sectioned at 5 µm. H&E staining was performed using standard techniques. For immunofluorescent staining, the rehydrated sections underwent heat‐mediated antigen unmasking with sodium citrate (Sigma–Aldrich) and quenching of endogenous peroxidase activity. The sections were then incubated at 4°C with the appropriate primary antibodies overnight. The list of antibodies used is in Table 1.

**TABLE 1:** Primary antibodies.

Target	Origin	Company	Cat. No.
KRT6	Mouse	Abcam	ab18586
IL‐13	Rabbit	Thermo Fisher Scientific	BS‐0560R
IL‐33	Goat	R&D Systems	AF3625
KRT14	Guinea pig	OriGene	BP5009
Ki67	Rat	Invitrogen	14‐5689‐82
IL‐1α	Rabbit	Abcam	ab9614
TSLP	Rabbit	Abcam	ab188766
IL‐33	Goat	R&D Systems	AF3626
PAR2	Rabbit	Abcam	ab180953
KLK5	Rat	R&D Systems	MAB7236
NFATc1	Rabbit	Thermo Fisher Scientific	PA5‐90432
EGF	Rabbit	Abcam	ab9695
EGFR	Rabbit	Abcam	ab52894
Lyvel	Rabbit	Abcam	ab14917
CD3e	Hamster	Thermo Fisher Scientific	14‐0031‐82
Type I collagen	Rabbit	Abcam	ab34710
Lyvel	Goat	R&D Systems	AF2125
CD4	Rat	R&D Systems	MAB554

H&E and immunofluorescent staining slides were evaluated with brightfield or fluorescent microscopy and scanned using a Mirax slide scanner (Carl Zeiss). Staining was visualised using a Pannoramic Viewer (3DHISTECH Ltd.). The epidermal area was quantified in H&E‐stained tail cross‐sections by measuring the ratio of dark stained epidermis within the total tissue area using MetaMorph Offline software (Molecular Devices) with a minimum of four HPFs per slide by two blinded reviewers. For mice tail cross‐sections, multiple HPFs were quantified covering up to 80% of tail cross‐section and for human biopsies the whole biopsy area was covered. Cell counts were quantified in immunofluorescent‐stained tail cross‐sections by counting the cells with positive staining. The protein‐expressing area was quantified as a ratio of the area of positively stained epidermis or dermis within a fixed threshold to total tissue area using MetaMorph Offline software with a minimum of four HPFs per slide by two blinded reviewers.

### 6.6 RNAseq

RNAseq was performed in collaboration with the Integrated Genomics Operation (IGO) Core Facility at MSK. Four pairs of frozen clinical lymphedema/normal skin biopsy specimens were submitted to the IGO. The ribodepletion method was used for RNAseq. mRNA expression was standardised and analysed by the IGO. Standardised expression for each molecule was assessed, and data are presented as *Z*‐scores.

### 6.7 Single‐cell transcriptome sequencing

Single‐cell suspensions were stained with Trypan blue, and the Countess II Automated Cell Counter (Thermo Fisher Scientific) was used to assess cell number and viability. After quality control, cells were loaded onto Chromium Next GEM Chip G (PN‐1000120; 10X Genomics), and GEM generation, cDNA synthesis, cDNA amplification and library preparation of 8000–12 000 cells were performed using the Chromium Next GEM Single Cell 3′ kit (v3.1, PN‐1000268; 10X Genomics) according to the manufacturer's instructions. cDNA amplification included 11 cycles, and 37–127 ng of the material was used to prepare sequencing libraries with 10–16 cycles of PCR. Indexed libraries were pooled equimolarly and sequenced on a NovaSeq 6000 in a PE28/88 run using the NovaSeq 6000 S4 Reagent kit (200 cycles; Illumina, Inc.). An average of 23 000 paired reads were generated per cell.

Raw sequencing data were demultiplexed and mapped to the reference genome GRCh38‐20 using Cell Ranger (v. 6.1.2; 10X Genomics). Filtered datasets (HDF5 files) from Cell Ranger for each of the paired patient samples representing lymphedema‐affected and unaffected limbs were analysed with Scanpy^89^ using a donor‐aware paired‐tissue computational workflow. Low‐quality cells were filtered out according to the expressed gene number, number of unique molecular identifiers (UMIs), mitochondrial percentage and ribosomal percentage. Specifically, the minimum number of unique genes per cell was 200. The minimum number of UMIs per cell was 500. Cells with a maximum of 50% ribosomal percentage were included. Cells with a maximum of 20% mitochondrial genes were included. Log‐normalised data underwent linear dimensional reduction through principal component analysis (PCA), followed by integration with Harmony.^90^ The top 3000 variable genes were used for PCA. The first 30 principal components were used for Harmony integration, Leiden cell clustering (resolution .5) and uniform manifold approximation and projection (UMAP) dimensional reduction.

Cell populations were identified using CellTypist^91^ with the Adult Human Skin model for automated cell type annotation. The keratinocyte population was identified as the cluster with a majority of cells demonstrating up‐regulation of keratinocyte markers based on the CellTypist classification. Expression of specific genes, including F2RL1 (PAR2), was queried of all cell cluster populations. Statistical comparisons between conditions were performed using paired *t*‐tests at the donor level to account for the paired tissue design. The keratinocyte population was identified based on this automated annotation. Expression of F2RL1 (PAR2) was quantified across all keratinocytes, with F2RL1‐positive cells defined as those with raw UMI counts greater than zero.

### 6.8 Real‐time PCR

Total RNA was extracted using TRIzol (Invitrogen) according to the manufacturer's instructions, and cDNA was prepared using Maxima™ H Minus cDNA Synthesis Master Mix (Thermo Fisher Scientific). Real‐time qPCR (qRT‐PCR; ViiA7; Life Technologies) was performed in duplicates using predesigned primer sets (Quantitect Primer Assays; Qiagen). Relative mRNA expression between groups was analysed by the delta‐delta Ct method and normalised to housekeeping genes β‐actin or GAPDH. Standardised expression for each molecule was assessed, and data are presented as *Z*‐scores.

### 6.9 Western blot

Clinical and mouse skin biopsies were frozen in liquid nitrogen, homogenised and lysed with a radioimmunoprecipitation assay lysis buffer containing a Halt™ Protease and Phosphatase Inhibitor Cocktail (Thermo Fisher Scientific). The lysates were centrifuged at 13 000×*g* for 10 min at 4°C, and protein concentration was measured using the BCA Protein Assay Kit (Thermo Fisher Scientific) according to the manufacturer's instructions. One to 20 µg of total protein was separated by NuPAGE™ 4–12% Bis–Tris gel (Thermo Fisher Scientific) and transferred onto PVDF membranes (Bio‐Rad). Membranes were blocked with 5% skim milk in TBS containing .1% Tween 20 (TBST) at room temperature for 1 h and incubated with antibodies against KRT6 (ab18586; Abcam), KRT16 (ab154361; Abcam), PAR2 (ab180953; Abcam), KLK5 (MAB7236; R&D Systems), IL13 (BS‐0560R; Thermo Fisher Scientific) and β‐actin (3700s; Cell Signaling Technology) in .5% skim milk in TBST at 4°C overnight. After washing three times with TBST, membranes were incubated with HRP‐conjugated secondary antibody in TBST at room temperature for 1 h. Next, the membranes were washed with TBST, and immune‐reactive bands were detected with ECL Western Blotting Substrate (Thermo Fisher Scientific). Protein expression was quantified with ImageJ software (National Institutes of Health) and normalised to housekeeping genes GAPDH or β‐actin.

### 6.10 ELISA

ELISA was performed using our published methods.^8^ In brief, tail skin tissue was harvested 1.5 cm distal to the surgical site and flash‐frozen in liquid nitrogen, and protein was extracted using a tissue extraction protein reagent (Thermo Fisher Scientific) mixed with phosphatase and protease inhibitor (Sigma–Aldrich). Approximately 20–30 mg of protein from each sample was analysed per the manufacturer's recommendations. The following ELISA kits were used: TSLP Mouse ELISA kit (EMTSLP; Thermo Fisher Scientific) and Mouse IL‐33 Quantikine ELISA kit (M3300; R&D Systems). All samples were assessed in triplicate.

### 6.11 Flow cytometry

Flow cytometry was performed to quantify inflammation in the mouse tails after tail surgery.^16^ In brief, single‐cell suspensions were obtained from a 1‐cm portion of the tail distal to the surgical site using a combination of mechanical dissociation and enzymatic digestion with a solution of DNase I, Dispase II, collagenase D and collagenase IV (all Roche Diagnostics) mixed in 2% FCS (Sigma–Aldrich). Cells were stained with combinations of the following fluorophore‐conjugated anti‐mouse monoclonal antibodies: rat CD45 (30‐F11; #103139), rat CD45 (30‐F11; #103116), rat CD11b (M1/70; #101228), Armenian hamster CD11c (N418; #117306), mouse CD207 (4C7; #144206), rat CD4 (GK1.5; #100408), Armenian hamster CXCR3 (CXCR3‐173; #126536), Armenian hamster CCR5 (HM‐CCR5; #107016), Armenian hamster CCR4 (2G12; #131214) and rat CCR8 (SA214G2; #150310) from BioLegend; and rat F4/80 (BM8; #25‐4801‐82) from eBioscience. In addition, DAPI viability stain was used on all samples to exclude dead cells. Single‐stain compensation samples were created using UltraComp eBeads™ (#01‐2222‐42; Affymetrix, Inc.). Flow cytometry was performed using a BD Fortessa flow cytometry analyser (BD Biosciences) with a BD FACS Diva, and data were analysed with FlowJo software (Tree Star).

### 6.12 Bone marrow transplantation

WT and PAR2KO recipient mice received two doses of 450 cGy gamma irradiation in a Gammacell Exactor 40 (Best Theratronics Ltd.) at 4 and .5 h before BMT. Bone marrow‐derived progenitor cells were collected from the femur of CD45.1 donor mice, and each recipient mouse received 1 million bone marrow‐derived progenitor cells through tail vein injection. At 2 months, we used double staining for CD45.1/CD45.2 to confirm the success of the BMT.

### 6.13 In vitro cell culture and treatment

H‐keratinocytes (PCS‐200‐011; ATCC) were cultured in dermal cell basal medium (PCS‐200‐030; ATCC) with a keratinocyte growth kit (PCS‐200‐040; ATCC). Keratinocytes were cultured with or without 10% lymphedema fluid in keratinocyte medium. 10 µg/mL ENMD1068, a PAR2 antagonist (ab141699; Abcam), was added once to the culture media at the same time as the lymphedema fluid treatment. TF (25 µM in DMSO) or DMSO alone was added to the culture media at the same time as the lymphedema fluid treatment. 150 µM AEBSF (A8456; Millipore Sigma) was added with or without 10% lymphedema fluid as a serine protease inhibitor. Cells were harvested 6 or 48 h after the treatment for RNA or protein extraction, respectively. Proliferation of keratinocytes, fibroblasts (CRL‐1658; ATCC) and lymphatic endothelial cells (C12217; PromoCell) was measured using a Vybrant® MTT Cell Proliferation Assay kit (V13154; Thermo Fisher Scientific). TF (in DMSO) or DMSO was added to the culture media a day after seeding for MTT assay and cultured for 24 h.

Primary m‐keratinocytes were collected from newborn pups. Back skin of newborn pups was collected and incubated with 4 mg/mL Dispase II in keratinocyte medium (C‐20011; PromoCell) for 16 h in the cold room. The skin was placed on 500 µL trypsin in a petri dish for 20 min on the shaker. After incubation, cells were collected with 2 mL of keratinocyte medium. Primary m‐keratinocytes were cultured with Lipofectamine (13778150; Life Technologies) and PAR2 siRNA (SI00074333; Qiagen) or control siRNA (1022076; Qiagen) in keratinocyte medium. After 3 days, cells were treated with or without 10% lymphedema fluid in keratinocyte medium. Cells were harvested 6 or 48 h after the treatment for RNA extraction or immunofluorescence staining, respectively.

### 6.14 Treatments

A topical formulation of 2 mM baicalein and 27 mg/mL TF dissolved in Aquaphor (Beiersdorf) was developed in collaboration with the MSK Research Pharmacy Core Facility. Control animals were treated with Aquaphor alone. The treatment was applied once daily for 2 or 4 week to the tail region distal to the zone of lymphatic skin excision or for 8 week to the footpad.

Mice were administered monoclonal antibody against mouse IL‐1α (5 µg/g/dose; clone ALF‐161; Bio X Cell) or isotype control antibodies (Bio X Cell) intraperitoneally for 4 week starting 2 week after surgery.

### 6.15 Lymph sample preparation for proteomics

The total protein concentration for lymph samples was determined using the Micro BCA™ Protein Assay Kit (#23235; Thermo Fisher Scientific). Equal protein aliquots (20–50 µg) were reduced with 10 mM TCEP.HCl (Thermo Fisher Scientific) in 50 mM ammonium bicarbonate buffer, pH 8.5, for 40 min at room temperature. The reduced proteins were further alkylated with 50 mM iodoacetamide solution for 50 min at room temperature. Three different enzymes were used for ‘in‐solution’ digestion in 50 mM ammonium bicarbonate buffer, pH 8.5, for 12 h at 37°C: endoproteinase Lys‐C (1:40 enzyme to protein ratio), trypsin (1:40 enzyme to protein ratio) and Glu‐C (1:20 enzyme to protein ratio). The digestion was quenched with .5% acetonitrile and 1.5% formic acid. Processed peptides were then extracted through a 10‐kDa molecular weight cutoff using 10‐kDa centrifugal filter units by spinning at 10 000×*g* for 15 min in a microcentrifuge. The peptide mixture, extracted from all enzymatic digestions, was desalted on C18 Prep clean columns before high‐resolution nanoflow liquid chromatography tandem mass spectrometry (nanoLC‐MS/MS).

### 6.16 Nanoflow liquid chromatography tandem mass spectrometry

The endogenous processed peptides from the human mesenteric lymph and the tryptic digests of mouse mesenteric lymph samples were analysed on a Q‐Exactive HF quadrupole orbitrap mass spectrometer (Thermo Fisher Scientific) coupled to an Easy nLC 1000 UHPLC (Thermo Fisher Scientific) through a nanoelectrospray ion source. The mass spectrometer was operated using a published protocol describing data‐dependent acquisition and positive ionisation mode.^92^, ^93^

### 6.17 Protein identification

Raw files from each technical and biological replicate were filtered, de novo sequenced and assigned with protein ID using Peaks X software (Bioinformatics Solutions) by searching against the reviewed human Swiss‐Prot database (March 2020; 205 500 entries). The search parameters were applied for label‐free quantification (LFQ) analysis: trypsin, Lys‐C and GluC restriction enzymes and two allowed missed cleavages at one or both peptide ends. The parent mass tolerance was set to 15 ppm using monoisotopic mass, and the fragment ion mass tolerance was set to .06 Da. Carbamidomethyl cysteine (+57.0215 on C) was specified in PEAKS as a fixed modification. Methionine, lysine, proline, arginine, cysteine and asparagine oxidations (+15.99 on CKMNPR); deamidation of asparagine and glutamine (NQ‐0.98); and pyro‐Glu from glutamine (Q‐18.01 N‐term) were set as variable modifications. Data were validated using the false discovery rate (FDR) method built in PEAKS X, and protein identifications were accepted with a confidence score (−10lgP) > 15 for peptides and (−10lgP) > 15 for proteins; a minimum of one peptide per protein was allowed after the data were filtered for a <1.5% FDR for peptides and a <2% FDR for proteins identifications (*p* < .05).

LFQ methods (spectral counting [MS/MS] and precursor MS1 intensity) were used to analyse and contrast the proteomic profiles of lymph from each patient. LFQ based on the precursor intensity was performed using the quantification algorithm supported by the PEAKS Q module (version X; Bioinformatics Solutions). The relative protein abundance was displayed as heat maps after normalisation of the corresponding averaged areas (abundances) with respect to the total ion current.^94^

### 6.18 Statistical analysis

Statistical analyses were performed using Prism 9.0.2 software (GraphPad). Samples were assessed for normal distribution using the Shapiro–Wilk test. Normally distributed clinical samples were analysed using a paired Student's *t*‐test. Comparisons of multiple groups or time points were performed using an unpaired Student's *t*‐test or Mann–Whitney test or a one‐way or two‐way ANOVA with multiple comparisons using Tukey's multiple comparison test. Data are presented as mean ± standard deviation unless otherwise noted, with *p* < .05 considered significant. For all plots, each dot represents one animal or patient unless noted otherwise.

## AUTHOR CONTRIBUTIONS

H. J. Park, R. P. Kataru and B. J. Mehrara conceived the concept and designed the research studies. H. J. Park, R. P. Kataru, L. Santambrogio, S. Pal and X. Chen conducted the experiments. H. J. Park, J. E. Baik, C. C. Clement, J. Shin, G. D. García Nores, A. Stull‐Lane, S. Pal and X. Chen acquired the data. H. J. Park, R. P. Kataru, G. D. García Nores, J. E. Baik, C. C. Clement, E. Riedel, L. Santambrogio, A. Stull‐Lane, X. Chen, A. J. Book, T. L. Chaunzwa, G. E. Hespe and B. J. Mehrara analysed data. H. J. Park, J. Shin, E. M. Encarnacion, M. G. Klang, M. Coriddi, J. H. Dayan and B. J. Mehrara provided the reagents and clinical skin biopsies. H. J. Park, X. Chen, A. J. Book, R. P. Kataru and B. J. Mehrara wrote the manuscript.

## CONFLICT OF INTEREST STATEMENT

B. J. Mehrara is the recipient of investigator‐initiated research grants from PureTech, Pfizer and Regeneron corporations and has received royalty payments from PureTech, Mediflix and Elsevier. J. H. Dayan is a consultant for Stryker Corporation and a director of Welwaze Medical LLC. No other disclosures were reported.

## ETHICS STATEMENT

All clinical procedures were approved by the Institutional Review Board (IRB protocol 17–377) at Memorial Sloan Kettering Cancer Center (MSK). Informed consents were obtained from all participants. All animal studies were approved by the Institutional Animal Care and Use Committee (IACUC) at MSK (protocol 06‐08‐018). The MSK IACUC adheres to the National Institutes of Health Public Health Service Policy on Humane Care and Use of Laboratory Animals and operates in accordance with the Animal Welfare Act and the Health Research Extension Act of 1985.

## CODE AVAILABILITY

The code that supports the single‐cell RNA sequencing results is openly available in GitHub at https://github.com/SecondBook5/lymphedema_keratinocyte_par2.

## Supporting information



Supporting Information

## Data Availability

Single‐cell RNA‐seq (GSE316723) and bulk RNA‐seq (GSE302118) data were deposited in the Gene Expression Omnibus (GEO). The data are available from the corresponding authors upon reasonable request.
