# Supplementary material for: Critical role of keratinocytes and protease‐activated receptor 2 in secondary lymphedema development
Source: Clin Transl Med. 2026 Jun 1;16(6):e70682. doi: 10.1002/ctm2.70682 (PMC13239967; doi:10.1002/ctm2.70682)
Supplement: Supplementary file 1 — Supporting Information [file CTM2-16-e70682-s001.docx]

**Figure S1. Expression of keratinocyte growth factors and inflammatory cytokines are upregulated in lymphedema.** **(A)** Relative mRNA expression by qPCR of KRT6, KRT14, and KRT16 in normal and LE skin biopsies from patients with unilateral BCRL (*n* = 10). mRNA expression was normalized to β-actin expression. Each box is representative of one patient. *P < 0.05, **P < 0.01. P values were calculated by paired Student’s *t* test. Fold change comparing LE versus normal skin biopsy is shown in parentheses. **(B)** Immunofluorescent analysis of lymphedema skin biopsies with each antibody (Ab) and respective negative controls without primary antibodies. Scale bar: 50 µm. **(C)** Representative immunofluorescent images (left) and quantification (right) of the EGF, EGF receptor (EGFR), IL-1α, and NFATc1 areas in normal and LE skin biopsies. Scale bar: 50 µm. Each circle represents the average quantification of three HPF views for each patient (*n* = 10–15). *P < 0.05, **P < 0.01. P values were calculated by paired Student’s *t* test.

**Figure S2. Keratinocyte-specific expression of F2RL1/PAR2 and state-resolved analysis in lymphedematous skin. (A)** Heatmap depicting z-score–normalized bulk RNA expression of F2RL1/PAR2-associated genes at the individual donor level (left, n = 4) and pooled across samples (right). **(B)** Subclustering of keratinocytes identified 11 distinct transcriptional states. **(C)** Marker-based annotation of keratinocyte states revealed basal, differentiated, junctional, stress-response, cycling, mesenchymal-like, and minor contaminant populations. **(D)** Donor-level heatmap of keratinocyte state abundance shifts in LE relative to paired normal skin, with the differentiated keratinocyte population (KC3) showing the greatest enrichment among epithelial states. **(E)** Corresponding donor-level heatmap of shifts in the fraction of F2RL1-positive cells within each keratinocyte state in LE relative to paired normal skin, indicating that F2RL1 expression varies across keratinocyte states rather than uniformly across all keratinocytes.

**Figure S3. PAR2KO decreases fibrosis, CD4^+^ cell infiltration, and hyperkeratosis after lymphatic injury.** **(A)** Representative gross images of tails from WT and PAR2KO mice harvested 6 wk after tail skin and lymphatic excision. **(B)** Representative H&E staining and immunofluorescent images of collagen LYVE1 staining in the lymphedema tail skin of WT and PAR2KO mice harvested 2 wk and 6 wk after tail skin and lymphatic excision. Scale bars: 500 µm (H&E) and 200 µm (immunofluorescence). **(C)** Representative immunofluorescent images of collagen I, LYVE1, and CD4 staining in the lymphedema tail skin of WT and PAR2KO mice harvested 6 wk after tail skin and lymphatic excision. Scale bar: 100 µm. **(D)** Representative immunofluorescent images of the KLK5, PAR2, and NFATc1 areas in the tail skin of WT and PAR2KO mice. Scale bar: 100 µm. **(E)** Quantification of KLK5, PAR2, and NFATc1 in the tail skin of WT and PAR2KO mice. Each circle represents the average quantification of three HPF views for each mouse (*n* = 5). **P < 0.01. P values were calculated by Mann–Whitney test. **(F)** Quantification of the epidermal area in the tail skin of WT and PAR2KO mice. Each circle represents the average quantification of three HPF views for each mouse (*n* = 5). *P < 0.05. P values were calculated by Mann–Whitney test. **(G)** Quantification of KRT6, Ki67, TSLP, and IL-33 in the tail skin of WT and PAR2KO mice. Each circle represents the average quantification of three HPF views for each mouse (*n* = 5). **P < 0.01. P values were calculated by Mann–Whitney test. **(H)** Representative western blots of KLK5 and PAR2 in the lymphedema tail skin of WT and PAR2KO mice. **(I)** Representative flow cytometry of LCs and Th2 cells from the tail skin and draining lymph nodes (LN) of WT and PAR2KO mice.

**Figure S4. Keratinocyte specific PAR2 expression is important for lymphedema development.** **(A)** Quantification of CD45.1 and CD45.2 populations in chimeric mice 2 mo after BMT (*n* = 6–9). **(B)** Representative gross images of WT→WT and WT→PAR2KO mice. **(C)** Representative gross images of PAR2^fl/fl^ and PAR2^cko^ mice. **(D and E)** Quantification of epidermal thickness, TSLP, and IL-33 in WT→WT and WT→PAR2KO mice (D) and PAR2^fl/fl^ and PAR2^cko^ mice (E). Each circle represents the average quantification of three HPF views for each mouse (*n* = 6). **P < 0.01, ***P < 0.001. P values were calculated by Mann–Whitney test. **(F and G)** Representative flow cytometry of LCs and Th2 cells from the tail skin and draining lymph nodes (LN) of WT→WT and WT→PAR2KO mice (F) and PAR2^fl/fl^ and PAR2^cko^ mice (G).

**Figure S5. Low-dose and early treatment of TF reduces lymphedema development. (A)** Representative immunofluorescent images of collagen I, LYVE1, and CD4 staining in tail skin harvested from mice treated with vehicle (control) or TF once daily for 4 weeks starting 2 weeks after tail skin and lymphatic excision. Scale bar: 100 µm. **(B)** Relative changes in tail volume over time in mice treated with control, high-dose TF, or low-dose TF for 4 weeks starting 2 weeks after tail skin and lymphatic excision. Each circle represents the average measurement for each mouse (*n* = 7). *P < 0.05, **P < 0.01. P values were calculated by two-way ANOVA with multiple comparisons. **(C)** Changes in tail volume over time in mice treated with control or TF for 2 wk starting 1 day after tail skin and lymphatic excision. Tail skin was harvested 2 wk after surgery. Each circle represents the average measurement for each mouse (*n* = 5). **P < 0.01. P values were calculated by two-way ANOVA with multiple comparisons. **(D)** Representative immunofluorescent images (left) and quantification (right) of NFATc1 and IL-1α staining in the tail skin from mice treated with control or TF. Scale bar: 100 µm. Each circle represents the average quantification of three HPF views for each mouse (*n* = 5). *P < 0.05, **P < 0.01. P values were calculated by Mann-Whitney test. **(E)** Western blot showed significant attenuation of IL13 expression in mouse tail skin with TF treatment (*n* = 6) compared with the control (*n* = 5). **P < 0.01. P values were calculated by Mann–Whitney test. **(F)** Representative immunofluorescent images (left) and quantification (right) of KRT6 and Ki67 staining in the tail skin from mice treated with control or TF. Scale bar: 100 µm. Each circle represents the average quantification of three HPF views for each mouse (*n* = 5). **P < 0.01. P values were calculated by Mann–Whitney test.

**Figure S6. Baicalein treatment reduces mouse tail edema and improves lymphatic drainage. (A)** Representative H&E-stained images of whole tail sections from mice treated with baicalein once daily for 4 weeks, beginning 2 weeks after tail skin and lymphatic excision, compared with vehicle-treated controls. Scale bars: 500 µm (low magnification, left) and 100 µm (high magnification, right). **(B)** Representative immunofluorescence images of LYVE1 staining (green) in tail skin collected from mice treated with vehicle (control) or baicalein once daily for 4 weeks, starting 2 weeks after tail skin and lymphatic excision. Scale bars: 200 µm (low magnification, left) and 100 µm (high magnification, right).

**Figure S7. Schematic overview of the study design and experimental workflow.** This schematic summarizes the overall study framework investigating the role of PAR2 signaling and Th2-associated inflammation in secondary lymphedema. Human tissue analyses (IHC, PCR, bulk RNA-seq, and scRNA-seq) identified increased expression of keratinocyte markers (KRT6, KRT14), TSLP, and IL-33, along with PAR2 involvement. Complementary animal studies demonstrated that Th2-mediated inflammation contributes to swelling, fibrosis, and keratinocyte activation. Mechanistic insights were further explored using multiple murine models, including PAR2-deficient, bone marrow chimeric, and keratinocyte-specific PAR2 models. Finally, topical therapeutic intervention studies targeting TSLP and PAR2 were conducted to evaluate potential treatment strategies.
